# The Impact of Working Beyond Retirement on Cognitive Function Among the Retirement-Transition Population

**DOI:** 10.3390/bs16091527

**Published:** 2026-08-30

**Authors:** Qianwen Sun, Longyi Huang, Sijie Cheng, Jialin Xu, Aijun Xu, Xuebin Qiao

**Affiliations:** 1 School of Health Economics and Management, Nanjing University of Chinese Medicine, Nanjing 210023, China; sqw@njucm.edu.cn (Q.S.); 20230944@njucm.edu.cn (L.H.); 20221025@njucm.edu.cn (S.C.); 202411034@njucm.edu.cn (J.X.); 2 Jiangsu Research Center for Major Health Risk Management and TCM Control Policy, Nanjing University of Chinese Medicine, Nanjing 210023, China

**Keywords:** working beyond retirement, delayed retirement, cognitive function, episodic memory, executive function, retirement-transition population, staggered difference-in-differences, CHARLS

## Abstract

With accelerating population aging and retirement-policy reforms, post-retirement work participation and its health effects have attracted increasing attention. Using data from the 2013–2020 waves of the China Health and Retirement Longitudinal Study (CHARLS), this study examined the effect of working beyond retirement on changes in cognitive function among the Retirement-Transition Population using a staggered difference-in-differences approach. Cognitive function was decomposed into episodic memory and executive function, and robustness checks were conducted using parallel trend tests and propensity score matching combined with difference-in-differences (PSM-DID). The results showed that working beyond retirement was positively associated with improved cognitive function. This effect was mainly observed in episodic memory, whereas its effect on executive function was limited. Heterogeneity analyses indicated that the cognitive effects were more pronounced among men and urban residents. Individuals without chronic diseases, without depressive symptoms, and with better self-rated health exhibited greater cognitive benefits. Among individuals with work-type change, those experiencing an agricultural-to-non-agricultural transition showed more pronounced cognitive benefits. The findings suggest that the cognitive effects of working beyond retirement are dimension-specific and heterogeneous across population groups. Future delayed retirement policies should recognize the health value of work participation, incorporate health benefits into policy considerations, and promote age-friendly employment through health assessment, job matching, and follow-up service mechanisms.

## 1. Introduction

As population aging accelerates worldwide, extending healthy life expectancy and promoting sustained social participation among older adults have become important issues in active aging strategies across countries. The United Nations Madrid International Plan of Action on Ageing identifies “older persons and development,” “advancing health and well-being into old age,” and “ensuring enabling and supportive environments” as three priority directions for addressing population aging, emphasizing that older adults should continue to participate in social development rather than merely be regarded as recipients of care (United Nations, 2002). The United Nations Decade of Healthy Ageing (2021–2030), led by the World Health Organization, further emphasizes the importance of maintaining older adults’ functional ability and social participation (World Health Organization, 2021). Meanwhile, retirement system reform and labor participation among older adults have gradually become central concerns in public policy. China provides an important context for examining this issue. In recent years, China has experienced substantial demographic changes, characterized by a continuous decline in the working-age population and an expansion of the older population, which have increased pressure on labor supply and old-age security systems. Data from the National Bureau of Statistics of China show that the working-age population aged 16–59 decreased from 922 million in 2012 (National Bureau of Statistics of China, 2019) to 858 million in 2024 (National Bureau of Statistics of China, 2025), while the old-age dependency ratio increased from 12.7% (National Bureau of Statistics of China, 2013) to 22.8% (Ministry of Civil Affairs of China, 2025) during the same period. In response to increasing pressures on labor supply and old-age security, China has continuously adjusted retirement-related policies in recent years. With the formal implementation of the Decision on Implementing a Gradual Delay in the Statutory Retirement Age (Standing Committee of the National People’s Congress, 2024) and the concurrent implementation of the Interim Measures for Implementing the Flexible Retirement System (Ministry of Human Resources and Social Security of China, 2025), China’s retirement system reform has moved from broad policy advocacy to substantive implementation. In this context, working beyond retirement has gradually emerged as an important social phenomenon receiving increasing attention amid retirement-system adjustments. Against this background, individuals undergoing the transition from working life to retirement have become an important population of interest, which this study defines as the Retirement-Transition Population. Compared with older adults who have already entered a stable retirement stage, the Retirement-Transition Population has distinct life-course characteristics. On the one hand, this population is at a critical stage of transition from occupational identity to retirement identity. During this period, work participation, social interactions, and lifestyles may undergo substantial changes. Continuing to work not only reflects economic participation but may also influence social role identity, psychological well-being, and cognitive function. On the other hand, this group is directly affected by delayed retirement policies, and their labor choices may reflect individual responses to changes in retirement-system arrangements. Therefore, focusing on the Retirement-Transition Population helps clarify how post-retirement work participation affects health outcomes from the intersection of life-course perspectives and institutional change. It also provides empirical evidence for promoting healthy aging amid ongoing retirement-system reforms.

Cognitive function is a core dimension of health in later life and is directly related to older adults’ activities of daily living and quality of life. Cognitive impairment is not a normal manifestation of cognitive aging, but rather a pathological condition. As population aging deepens, cognitive impairment has become a major public health issue threatening the quality of life of older adults in China. Evidence shows that among people aged 60 years and older in China, the prevalence of dementia is approximately 6%, the prevalence of mild cognitive impairment is approximately 15.5%, and the total number of affected individuals is approximately 53 million (L. Jia et al., 2020). Evidence from the Global Burden of Disease Study indicates that mortality from Alzheimer’s disease and other dementias in China increased substantially between 1990 and 2019, with its ranking among causes of death rising from tenth to fifth (Lv et al., 2023). Meanwhile, the total cost attributable to Alzheimer’s disease in China is projected to reach 11.9 trillion yuan by 2050 (J. Jia et al., 2018). As cognitive function declines, individuals’ quality of life decreases substantially (Landeiro et al., 2020). Therefore, identifying intervention strategies that can delay cognitive decline and protect cognitive function has become an important issue in achieving healthy aging.

Guided by the concept of “proactive health,” active aging strategies emphasize improving quality of life in later life by enhancing older adults’ autonomy and participation. The Chinese cultural tradition of “remaining productive in old age” further encourages older adults to continue contributing and participating in social activities within their capabilities. Working beyond retirement is not only an objective demand of the labor market, but also an important pathway through which older adults can realize self-worth. Can this practice of “remaining productive in old age” truly translate into cognitive health benefits? If so, in which specific dimensions of cognitive function are these benefits manifested? And what factors may account for differences in these effects? Answering these questions not only helps reassess the value of delayed retirement policies from the perspective of health benefits, but also provides new insights into individuals’ work participation behaviors and health consequences during the transition of retirement identity. It also provides new empirical evidence for advancing active aging strategies under the concept of proactive health.

Existing studies have examined the relationships among retirement, labor participation, and health in later life from multiple perspectives, but their conclusions remain inconsistent. One view holds that regular retirement benefits individual health by reducing work-related stress and increasing leisure time (Westerlund et al., 2010; Westerlund et al., 2009; Jokela et al., 2010; Mein et al., 2003; Lang et al., 2007), whereas continued work may bring additional pressure. Another view argues that delayed retirement can increase income and personal activity, thereby producing more favorable health outcomes (Sewdas et al., 2020; Shiba et al., 2017; Rose et al., 2023). To further examine the health effects of working beyond retirement, some scholars have focused on specific health indicators. For example, studies on bridge employment have found that, compared with full retirement, continuing to engage in some form of work after retirement may be associated with better physical and mental health outcomes (Zhan et al., 2009); other studies have found that the relationship between post-retirement work and mental health indicators such as depressive symptoms varies in direction and across groups (Y. Park et al., 2025). Beyond general health and mental health, cognitive function, as a core dimension of health in later life, has received extensive scholarly attention. Existing systematic reviews indicate that employment in later life is generally positively associated with cognitive function, although this association varies across studies and population groups (Takase et al., 2024). Some scholars have noted that, compared with prolonged housework or unemployment, extended full-time or part-time work is beneficial to cognitive function in later life, although this effect exhibits substantial population heterogeneity (Bertogg & Leist, 2023). A study from Japan found that continued employment after retirement is beneficial to cognition when accompanied by a change in employer (Mizuochi & Raymo, 2022). In addition, the effect of retirement on cognitive function is not entirely negative; at least in the early stage of retirement, it may be beneficial (Celidoni et al., 2017). International studies provide an important foundation for understanding the relationship between working beyond retirement and cognitive function, but this effect may exhibit different characteristics within China’s distinctive labor environment and social security context. Related studies in Chinese and broader East Asian contexts further reveal this complexity. A study from Taiwan found that paid work can protect cognitive function, whereas unpaid work is associated with a higher risk of cognitive impairment (Hsu, 2007). Studies based on Chinese data have found clear heterogeneity in the relationship between post-retirement work and mental health among older adults (Xie et al., 2021); another study using CHARLS data found that post-retirement work is associated with the risk of frailty (Sun et al., 2024). Meanwhile, studies on healthy working life expectancy and post-retirement work suggest that policy discussions on extending working life should fully consider health capacity, job quality, and individual differences (C. Li et al., 2024; Huang et al., 2025; Zaccagni et al., 2024). A review of these studies shows that, both internationally and domestically, scholarly understanding of the relationship between working beyond retirement and cognitive function has gradually deepened, moving from early concerns about whether an effect exists to examining heterogeneous effects under different conditions. Whether in terms of the role of occupational complexity confirmed by international studies or the differences in health status and institutional context revealed in Chinese studies, existing evidence suggests that the effect of working beyond retirement on cognitive function is neither uniformly beneficial nor uniformly harmful, but rather characterized by complex heterogeneity.

In summary, existing research has laid an important foundation for understanding the relationship between working beyond retirement and cognitive function. In cognitive function research, episodic memory and executive function are two core dimensions that play key roles in cognitive health in later life. Episodic memory is responsible for the retention and retrieval of past experiences, whereas executive function is responsible for the regulation and control of current behavior. However, few studies have specifically distinguished between these two dimensions; most treat cognitive function as a single overall indicator, making it difficult to reveal the specific mechanisms through which particular factors affect cognition. In research on working beyond retirement, scholars have developed a relatively clear consensus on its conceptual definition and have systematically explained its behavioral motivations from multiple perspectives, including economic goals, family support, health needs, and self-realization, thereby providing a solid theoretical basis for subsequent research.

However, a review of the existing literature reveals several limitations in studies on the relationship between working beyond retirement and cognitive function: (1) in terms of research subjects, most studies treat adults aged 60 and above as a single group and fail to focus on the retirement-transition population, which is the key group affected by delayed retirement policies; (2) in terms of research design, existing studies are mainly cross-sectional and lack support from longitudinal data, making it difficult to infer the long-term causal relationship between working beyond retirement and cognitive function; and (3) in terms of heterogeneity analysis, existing studies still pay insufficient attention to differences within the working-beyond-retirement population. On the one hand, it remains necessary to further examine effect differences across demographic characteristics such as gender and urban-rural residence, as well as health conditions; on the other hand, specific behavioral contexts such as whether their type of work changes have received limited attention, although these factors may be key entry points for revealing the cognitive effects of working beyond retirement.

To address these gaps, clarifying the impact of working beyond retirement on cognitive function in the context of ongoing retirement-system reform and active aging strategies in China not only deepens the understanding of the health effects of later-life work participation but also provides empirical evidence for evaluating the health implications of delayed retirement policies. Therefore, this study uses data from the China Health and Retirement Longitudinal Study (CHARLS) from 2013 to 2020, focuses on the retirement-transition population, divides cognitive function into episodic memory and executive function, applies a staggered difference-in-differences approach to identify the effect of working beyond retirement on cognitive function, incorporates propensity score matching to address sample selection bias, and further uses subgroup regressions to examine heterogeneous patterns across demographic characteristics, health condition, and specific working-beyond-retirement behaviors, thereby revealing the boundary conditions under which working beyond retirement exerts cognitive health effects. The potential marginal contributions of this study are threefold: first, by focusing on the retirement-transition population, a key group affected by delayed retirement policies, this study examines working beyond retirement within the context of China’s retirement-system adjustments and active aging, extending existing retirement research from comparisons between retirement statuses to an analysis of the retirement-transition process; second, by dividing cognitive function into episodic memory and executive function, it helps identify the specific cognitive domains affected by working beyond retirement; and third, by incorporating work-type change as a specific behavioral context, it contributes to a more refined understanding of the cognitive health benefits and boundary conditions of working beyond retirement and provides scientific evidence for the precise implementation of active aging strategies.

## 2. Theoretical Foundation and Research Hypotheses

### 2.1. Working Beyond Retirement and Cognitive Function

In this study, “working beyond retirement” is defined as continuing to engage in paid work or labor participation after reaching retirement age (Dingemans & Möhring, 2019). Based on cognitive reserve theory, this study examines the effect of working beyond retirement on cognitive function. Drawing on previous research on cognitive function, this study divides cognitive function into executive function and episodic memory to further reveal the cognitive effects of working beyond retirement.

Cognitive reserve theory was formally proposed by Yaakov Stern, a psychologist at Columbia University, in 2009 (Stern, 2009), and is used to explain why some individuals are able to maintain relatively normal cognitive function despite brain injury or neuropathological changes (Sunderaraman & Stern, 2019). Cognitive reserve refers to the neural resources accumulated through genetic factors and life experiences, such as educational attainment, occupational complexity, and leisure activities, which help the brain cope more effectively with cognitive impairment caused by aging or disease (Sunderaraman & Stern, 2019; Scarmeas & Stern, 2003). Unlike brain reserve theory, which is based on “hardware” indicators such as brain volume and neuronal quantity, cognitive reserve places greater emphasis on “software” factors such as intellectual stimulation and cognitive strategy selection, highlighting individual differences in cognitive performance (Stern et al., 2019). Among these factors, task complexity, decision-making demands, and problem-solving experiences embedded in occupational activities constitute important pathways through which individuals accumulate cognitive reserve during adulthood (Stern, 2009).

Existing international research further suggests that cognitive reserve can be accumulated through life-course experiences such as education and cognitively stimulating activities, and plays an important role in protecting cognitive health in later life (Livingston et al., 2024). At the same time, social relationships and social participation are also important factors affecting cognitive function, and higher cognitive reserve and richer social interactions help buffer the negative effects of risk factors such as social isolation and loneliness on cognitive function (Kuiper et al., 2016).

**Figure 1:**
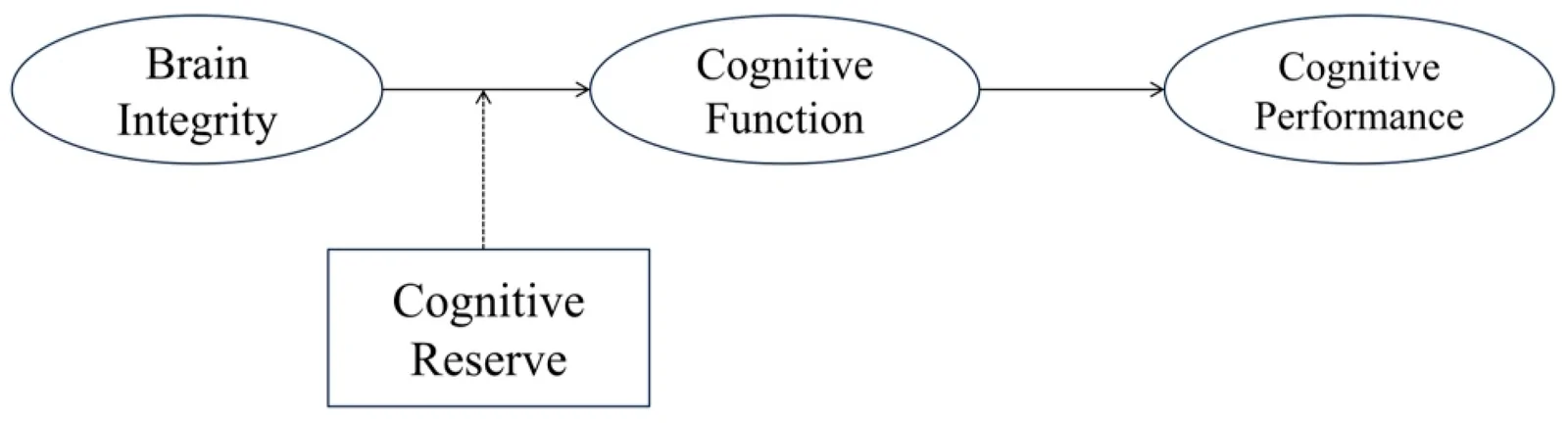
Basic model of cognitive reserve theory.

This theory provides important theoretical support for the present study. According to cognitive reserve theory, an individual’s brain integrity determines cognitive ability, which in turn affects cognitive performance, while cognitive reserve plays a moderating role in this process. Higher cognitive reserve enables individuals to maintain relatively normal cognitive function even in the presence of brain aging or pathological changes (Stern, 2009), as shown in Figure 1. Within this theoretical framework, the continuous stimulation brought about by working beyond retirement may continuously enhance cognitive reserve among the retirement-transition population. Specifically, compared with full retirement, continuing to work after retirement provides the cognitive system with various challenges and forms of training, which helps maintain or enhance cognitive reserve and thereby delay the cognitive decline. This theoretical perspective provides an important basis for understanding the cognitive protective effect of working beyond retirement in this study. Therefore, this study proposes the following hypothesis.

**Hypothesis** **1.** *Working beyond retirement has a positive effect on cognitive function*.

### 2.2. The Effects of Working Beyond Retirement on Different Cognitive Domains

Episodic memory primarily depends on the function of the medial temporal lobe and is relatively sensitive to lifestyle interventions (Dahan et al., 2020). Work processes require the retrieval and application of past information, such as recalling work procedures, and these activities continuously exercise the episodic memory system. For older adults who work beyond retirement, regular memory demands at work repeatedly stimulate relevant brain regions, helping maintain their structural integrity and function and thereby delaying the decline of episodic memory. Social interactions and emotional communication in work settings provide substantial support for memory. Social interactions such as communication with colleagues and contact with clients create numerous memory contexts, while the sense of achievement after completing tasks and the satisfaction of solving problems bring vivid emotional experiences. These contextual details and events form richer and more stable memory traces, allowing memory ability to be maintained more effectively.

Executive function primarily depends on the prefrontal cortex and its connections with subcortical structures, and mainly involves higher-order cognitive tasks such as planning, inhibition, and task switching. Specifically, multitasking, decision-making, and problem-solving in work environments are core application scenarios of executive function (Diamond & Ling, 2016). Multitasking at work requires individuals to switch flexibly between different tasks and allocate attention and time, a process that continuously activates the prefrontal system. When facing complex problems, individuals need to quickly formulate solutions and prepare for implementation, which depends on the immediate allocation of cognitive resources by executive function. Unlike episodic memory, the maintenance of executive function derives more directly from cognitive challenges, such as work-related problems and decisions, which provide regular training opportunities for the prefrontal system and keep it active during work. Therefore, working beyond retirement may help delay the decline of executive function in older adults by continuously activating the prefrontal system. Therefore, this study proposes the following hypotheses.

**Hypothesis** **1a.** *Working beyond retirement has a positive effect on episodic memory.*

**Hypothesis** **1b.** *Working beyond retirement has a positive effect on executive function.*

### 2.3. Work-Type Change and the Cognitive Effects of Working Beyond Retirement

Continuity theory was formally proposed by the American gerontologist Robert Atchley in 1989 and provides an important theoretical perspective for understanding how older adults adapt to aging (Atchley, 1989). According to continuity theory, older adults do not necessarily need to withdraw from the social environment, nor do they need to deliberately maintain highly active social engagement. Rather, they tend to continue familiar lifestyles, routines, and interpersonal relationships, which helps maintain self-identity and mental health and enables better adaptation to later life. Continuity in continuity theory is mainly reflected at two levels. Internal continuity mainly refers to psychological characteristics such as personality, values, and beliefs, which determine how individuals perceive their relationship with themselves and the world, whereas external continuity includes the continuation of social roles, daily activities, and patterns of interpersonal interaction. These two levels are intertwined and jointly influence older adults’ adaptation to later life.

According to continuity theory, working beyond retirement can be regarded as a continuation of previous occupational roles. For individuals who have long derived a sense of value from work, continuing to work after retirement helps maintain external continuity by preserving stable social roles and life rhythms, whereas for those who expect to enjoy leisure life, full retirement may better align with their internal continuity and fulfill their genuine inner needs. Notably, work-type change is closely related to continuity theory. In later discussions, Atchley further noted that continuity does not imply rigidity or immutability, but allows adaptive adjustments in external roles and activity patterns (Atchley, 1999). From this perspective, work-type change does not necessarily create difficulties. When individuals change their type of work, external continuity may change, but if such a transition brings higher levels of cognitive stimulation and social participation, it may instead produce positive adaptive effects. This theoretical perspective helps explain the heterogeneous outcomes observed among different groups in the process of working beyond retirement.

Furthermore, when individuals move from a familiar field to a new work environment, they often need to mobilize more cognitive resources to adapt to new demands, a process that may provide additional cognitive stimulation. Compared with continuing to engage in familiar tasks within the original work domain, work-type change imposes higher adaptive demands on the cognitive system. Individuals need to learn new work skills, establish new interpersonal relationships, and face new work challenges, which continuously activates brain regions such as the prefrontal cortex and hippocampus. Research has shown that cognitive challenge is an important factor in maintaining cognitive function, and familiar work content may not provide sufficient stimulation for cognitive function, whereas work-type change provides continuous novel stimulation (Oltmanns et al., 2017). Individuals need to reposition their roles within the work environment and establish new social connections, and this process of social adaptation helps maintain cognitive flexibility and contextual adaptability (Dajani & Uddin, 2015). Therefore, compared with individuals whose work content remains unchanged, those who experience work-type change may receive more diverse cognitive stimulation.

**Hypothesis** **2.** *The positive effect of working beyond retirement on cognitive function is mainly observed among those who have experienced work-type change*.

## 3. Materials and Methods

### 3.1. Data Source

The data used in this study were derived from the China Health and Retirement Longitudinal Study (CHARLS). CHARLS is conducted by the National School of Development at Peking University and aims to collect nationally representative micro-level data on individuals and households aged 45 years and above in China. The survey adopts a multistage stratified sampling method and covers 150 counties and 450 communities or villages across 28 provinces, autonomous regions, and municipalities in China. Information is collected through face-to-face interviews and structured questionnaires, covering multiple dimensions such as respondents’ demographic characteristics, family structure, health status, health service utilization, work and retirement, pension insurance, income, and consumption. The project conducted its national baseline survey in 2011 and has followed respondents every two years since then, forming multiple waves of longitudinal data that provide a high-quality empirical basis for studying issues related to population aging in China. This study used CHARLS longitudinal data from 2013 to 2020 to examine the relationship between working beyond retirement and cognitive function among the retirement-transition population.

### 3.2. Sample Selection

The study population consisted of the retirement-transition population. This group is at a critical stage in the transition from working life to retirement and faces the key life-course choice between full retirement and continued work, making it an ideal population for studying working beyond retirement.

To identify the treatment timing of working beyond retirement, strict sample selection was required to ensure that each individual could be observed throughout the complete transition from not working beyond retirement to retirement or working beyond retirement. Therefore, this study set the following age-based inclusion criteria for individuals: first, the baseline age criterion. At the time of the first survey, respondents had to be employed and had not yet reached the statutory retirement age. According to China’s current retirement system, the statutory retirement age is 60 years for men, while the retirement age for women varies by occupational status. Due to data limitations, women’s retirement age could not be directly determined. Following the approach adopted in previous studies (Feng et al., 2020), women with a bachelor’s degree or above were assumed to retire at age 55, while other women were assumed to retire at age 50. Second, the endline age criterion. At the time of the final survey, respondents had to be older than the statutory retirement age. To clarify these age-based criteria, consider an example: if a respondent participated in both the 2013 and 2020 surveys, his or her age in 2020 had to be over 60 years, and accordingly, his or her age in 2013 should have been between 54 and 59 years. This design ensured that individuals fully experienced the age transition into retirement during the observation period, allowing the timing of working beyond retirement to be identified. On the basis of these age criteria, individuals’ work status in the year when they first exceeded the statutory retirement age was used to determine whether they had engaged in working beyond retirement. If an individual was working in that year, he or she was defined as having worked beyond retirement; if not, he or she was defined as fully retired.

According to the above criteria, this study initially identified 4720 individuals who met the age requirements across four waves of CHARLS data. On this basis, to further ensure sample validity, the following exclusions were made: first, individuals who were not working at baseline were excluded, because accurately observing the transition from employment to retirement or working beyond retirement required respondents to be employed at baseline, resulting in the exclusion of 598 individuals. Second, individuals with missing cognitive function data in any of the four survey waves were excluded, resulting in the exclusion of 1922 individuals. Third, boundary cases were addressed by excluding individuals who had just reached the retirement age at the survey time point, because it was difficult to accurately determine whether they should be classified as working beyond retirement in that year, resulting in the exclusion of 864 individuals.

**Figure 2:**
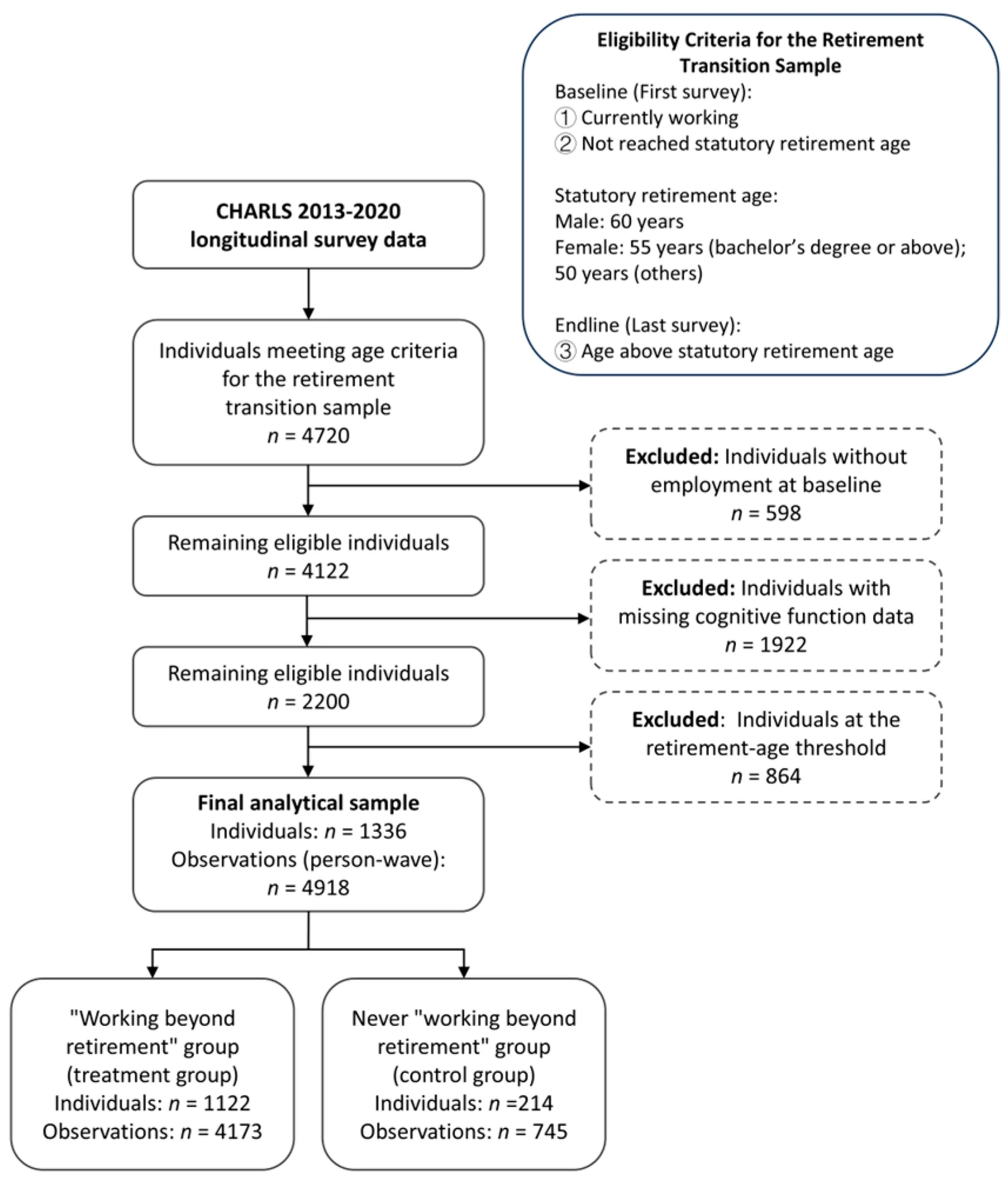
Flowchart of the study sample selection process.

After the above screening procedures, this study ultimately obtained 1336 valid individuals, comprising 4918 person-wave observations. The detailed sample selection process is presented in Figure 2. Among them, the treatment group, consisting of individuals who had worked beyond retirement, included 4173 observations, while the control group, consisting of individuals who had never worked beyond retirement, included 745 observations. For the small number of missing values in the control variables, this study used multiple imputation by chained equations to maximize the retention of sample information.

### 3.3. Variable Selection and Description

#### 3.3.1. Independent Variable

Labor participation in this study was measured based on a comprehensive assessment of multiple items in the CHARLS questionnaire, following previous research practices (Wan et al., 2021). The measurement included the following items: whether respondents had engaged in agricultural production or business activities for more than 10 days in the past year; whether they had performed paid work in the past week; whether they had a job but were temporarily on leave; whether they could return to their original position at a definite time or within six months; whether they had worked after completing retirement procedure; and whether they were currently engaged in small income-generating recreational work. If all of these items were answered “no,” the respondent was classified as not currently working; if any item was answered “yes,” the respondent was classified as currently working.

Based on the above classification of labor participation and the individual’s retirement-age threshold, this study operationalized the variable of working beyond retirement as follows (Cheng, 2014): if an individual had exceeded the statutory retirement age and was currently working in survey wave *t*, the working-beyond-retirement variable for that wave was assigned a value of 1. If an individual had not yet exceeded the statutory retirement age in survey wave *t*, or had exceeded the retirement age but was not currently working, the working-beyond-retirement variable for that wave was assigned a value of 0.

**Figure 3:**
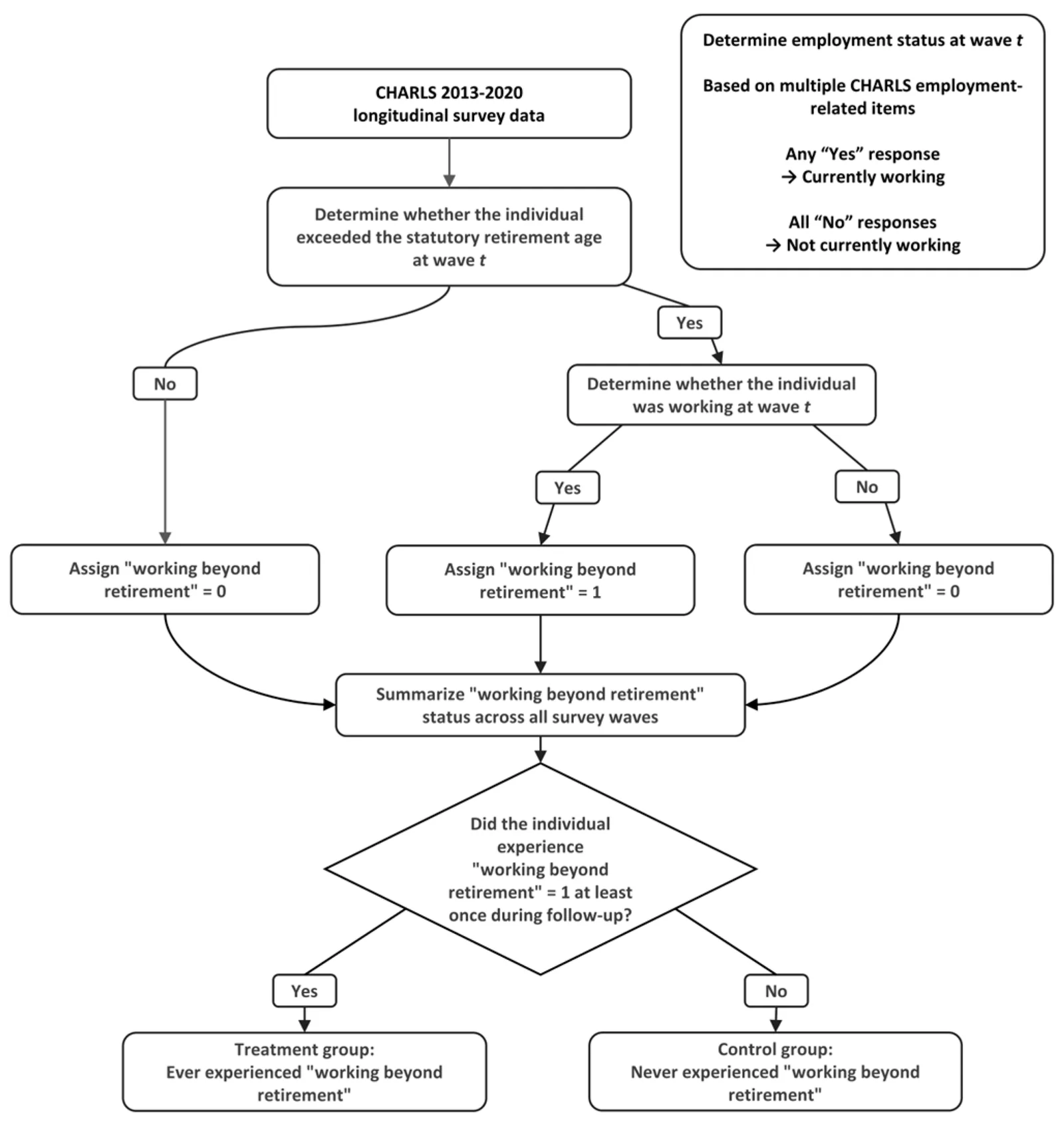
Flowchart of the construction of the working-beyond-retirement variable.

At the individual level, those who experienced working beyond retirement at least once during the observation period were classified into the treatment group, whereas those who never experienced working beyond retirement in any survey wave during the observation period were classified into the control group. To further illustrate the identification process of the working-beyond-retirement variable and the rules for individual grouping, a variable construction flowchart was developed (Figure 3).

#### 3.3.2. Outcome Variables

Following the CHARLS measurement framework, cognitive function was captured from two dimensions: episodic memory and executive function (Lin et al., 2022). Episodic memory was assessed using immediate and delayed word recall tasks. During the survey, interviewers read ten Chinese words in random order, after which respondents were asked to recall them immediately, and the number of correctly recalled words was recorded; after several minutes, respondents were asked to recall the same words again, and the number of correctly recalled words in delayed recall was recorded. Each correctly recalled word was assigned 0.5 points (Guo & Zheng, 2023), and the episodic memory score was calculated as the sum of the immediate and delayed recall scores, ranging from 0 to 10.

Executive function was assessed using the Telephone Interview for Cognitive Status (TICS) and a visuospatial construction task. The TICS included an orientation test, which required respondents to correctly report the date, including year, month, and day, the day of the week, and the season, as well as a calculation test, which required respondents to subtract 7 from 100 consecutively for five trials (M. Li et al., 2022). The score for this component was the total number of correct answers, ranging from 0 to 10. In the visuospatial construction task, respondents were asked to observe and copy a figure consisting of two overlapping pentagons, with 1 point assigned for successful completion and 0 points assigned for failure (J. Li et al., 2017). The executive function score was calculated as the sum of these two components, ranging from 0 to 11. The overall cognitive function score was calculated as the sum of the episodic memory and executive function scores, ranging from 0 to 21.

#### 3.3.3. Control Variables

This study controlled for a series of covariates, including demographic variables such as gender, age, educational attainment, marital status, residence, smoking status, and drinking status. Self-reported health variables included depressive symptoms, chronic disease, self-rated health, and bodily pain. Social characteristic variables included medical insurance, pension insurance, and social participation. The definitions and coding of all variables are presented in Table 1.

### 3.4. Statistical Model Specification

Based on the processed data, this study used Stata 17.0 (StataCorp LLC, College Station, TX, USA) to conduct a difference-in-differences (DID) analysis. Since the conventional DID approach can only estimate the effect of an intervention occurring at a single time point, whereas the timing of working beyond retirement varied across individuals in the study sample, this study adopted a staggered DID model (Goodman-Bacon, 2021), as shown in Equation (1).

(1) $$Y_{it}=\beta +\alpha {(Work}_{it}\times {RetireAge}_{it})+\delta X_{it}+\lambda_{i}+\nu_{t}+\varepsilon_{it}$$

In Equation (1), $Y_{it}$ denotes the cognitive function score of individual i in year t, including the two dimensions of episodic memory and executive function. The key independent variable $Work_{it}\times RetireAge_{it}$ indicates whether individual i was in the state of working beyond retirement in year t. Specifically, $Work_{it}$ indicates whether individual i was working in year t. If the individual was working, $Work_{it}=1$; otherwise, $Work_{it}=0$. $RetireAge_{it}$ indicates whether individual i had exceeded the statutory retirement age in year t. If the individual’s age exceeded the statutory retirement age, $RetireAge_{it}=1$; otherwise, $RetireAge_{it}=0$. α represents the average treatment effect of working beyond retirement on cognitive function. β is the constant term, and $\lambda_{i}$ represents individual fixed effects, which control for time-invariant unobserved factors at the individual level. $\nu_{t}$ represents time fixed effects, which control for common shocks affecting all individuals due to changes in the macro environment. $X_{it}$ denotes a series of time-varying individual-level control variables, including demographic characteristics such as age, marital status, and residence; socioeconomic security variables such as pension insurance and medical insurance; health behaviors such as smoking and drinking; health status variables such as chronic disease, self-rated health, and depressive symptoms; and social participation, while $\varepsilon_{it}$ is the error term. By simultaneously controlling for individual fixed effects and time fixed effects, this model can control for time-invariant individual-level unobserved factors and common shocks across years, thereby reducing omitted variable bias to some extent. It should be noted that working beyond retirement is not randomly assigned. Whether individuals continue working after reaching the statutory retirement age may still be influenced by unobserved or difficult-to-measure factors, including changes in health status, functional ability, household economic pressure, employment opportunities, and socioeconomic conditions.

In addition, to examine the reliability of the estimated results, this study further conducted robustness checks using parallel trend tests, propensity score matching, excluding early-retirement samples, and excluding individuals with mild cognitive impairment at baseline, and employed subgroup regressions to investigate group differences in the effect of working beyond retirement on cognitive function.

## 4. Results

### 4.1. Descriptive Statistics

Table 2 reports the descriptive statistics of the demographic characteristics and key variables of the study sample at baseline. The full sample included 1336 eligible individuals from the retirement-transition population, of whom 1122 were in the treatment group, accounting for 84.0%, and 214 were in the control group, accounting for 16.0%.

In terms of age distribution, the mean baseline age of the full sample was 52.10 years, and the age distributions of the treatment and control groups were relatively similar. Regarding gender composition, men accounted for 51.05% and women for 48.95% of the full sample, indicating a relatively balanced gender distribution. In terms of educational attainment, the full sample was mainly composed of individuals with junior middle school education, accounting for 31.96%, and primary school education, accounting for 31.74%. In the control group, the proportion of individuals with high school education or above reached 29.91%, which was higher than that in the treatment group at 18.54%, indicating that individuals with higher educational attainment accounted for a larger proportion of the control group. Regarding marital status, 96.63% of the full sample were partnered, and the difference between the treatment and control groups was relatively small. In terms of residence, the urban–rural distribution differed between the two groups: urban residents accounted for as high as 71.03% of the control group. By contrast, rural residents accounted for 63.73% of the treatment group, suggesting that rural residents accounted for a relatively higher proportion of those working beyond retirement. In terms of social security, the coverage rate of pension insurance in the full sample was only 4.27%, and the coverage rate was higher in the control group than in the treatment group. By contrast, medical insurance coverage was generally high, reaching 96.48% in the full sample, with only a small difference between the two groups. Regarding health status, the prevalence of chronic disease in the full sample was 64.75%, and the rates were relatively similar between the treatment and control groups. In terms of depressive symptoms, the mean score of the full sample was 6.77, and the average levels were largely consistent between the two groups. Regarding self-rated health, most respondents rated their health as fair, and the distributions were generally similar between the treatment and control groups. In terms of health behaviors, the drinking rate in the control group was 44.39%, and both the drinking and smoking rates were slightly higher in the control group than in the treatment group. Regarding social participation, the participation rate was higher in the control group than in the treatment group, suggesting that social participation may be associated with individuals’ choice to work beyond retirement.

### 4.2. Baseline Regression Results

Table 3 reports the difference-in-differences estimation results for the effect of working beyond retirement on cognitive function among the retirement-transition population. The model adopts a two-way fixed effects specification, controlling for both individual fixed effects and time fixed effects, in order to eliminate the influence of time-invariant individual heterogeneity and common time trends.

Regarding the core explanatory variable, working beyond retirement has a significant positive effect on overall cognitive function (α = 0.198, *p* = 0.031). This indicates that, compared with the fully retired group, the working-beyond-retirement group experienced a significantly greater change in cognitive function before and after retirement, with an average additional increase of 0.198 points in cognitive function score. This result supports Hypothesis 1, namely that working beyond retirement has a positive effect on cognitive function among the retirement-transition population. Further dividing cognitive function into the two dimensions of episodic memory and executive function, the results show that working beyond retirement has a significant positive effect on episodic memory (α = 0.158, *p* = 0.006), whereas its effect on executive function does not reach statistical significance (α = 0.039, *p* = 0.553). This finding suggests that the cognitive effects of working beyond retirement exhibit some dimension-specific patterns, with positive effects mainly observed in episodic memory, whereas the effect on executive function is relatively limited. Therefore, Hypothesis 1a is supported, whereas Hypothesis 1b is not supported. The calculation of standardized effect sizes further showed that the effects of working beyond retirement on overall cognitive function, episodic memory, and executive function were 0.071, 0.096, and 0.021 standard deviations, respectively. Although the effect sizes were relatively small, these findings may still have important population health implications, considering that cognitive function is a health indicator that gradually changes with aging.

The estimated results for the control variables are generally consistent with existing studies. In terms of age, compared with the 45–54 age group, individuals aged 60 and above show significant declines in overall cognitive function and episodic memory, which is consistent with the general pattern of cognitive aging. In terms of educational attainment, individuals with high school education or above perform significantly better in executive function than those with below primary school education. In terms of health status, depressive symptoms are significantly negatively associated with overall cognitive function, episodic memory, and executive function, while chronic disease is significantly negatively associated with executive function. In terms of social participation, individuals who participate in social activities have significantly better overall cognitive function than those without social participation.

### 4.3. Parallel Trend Test

**Figure 4:**
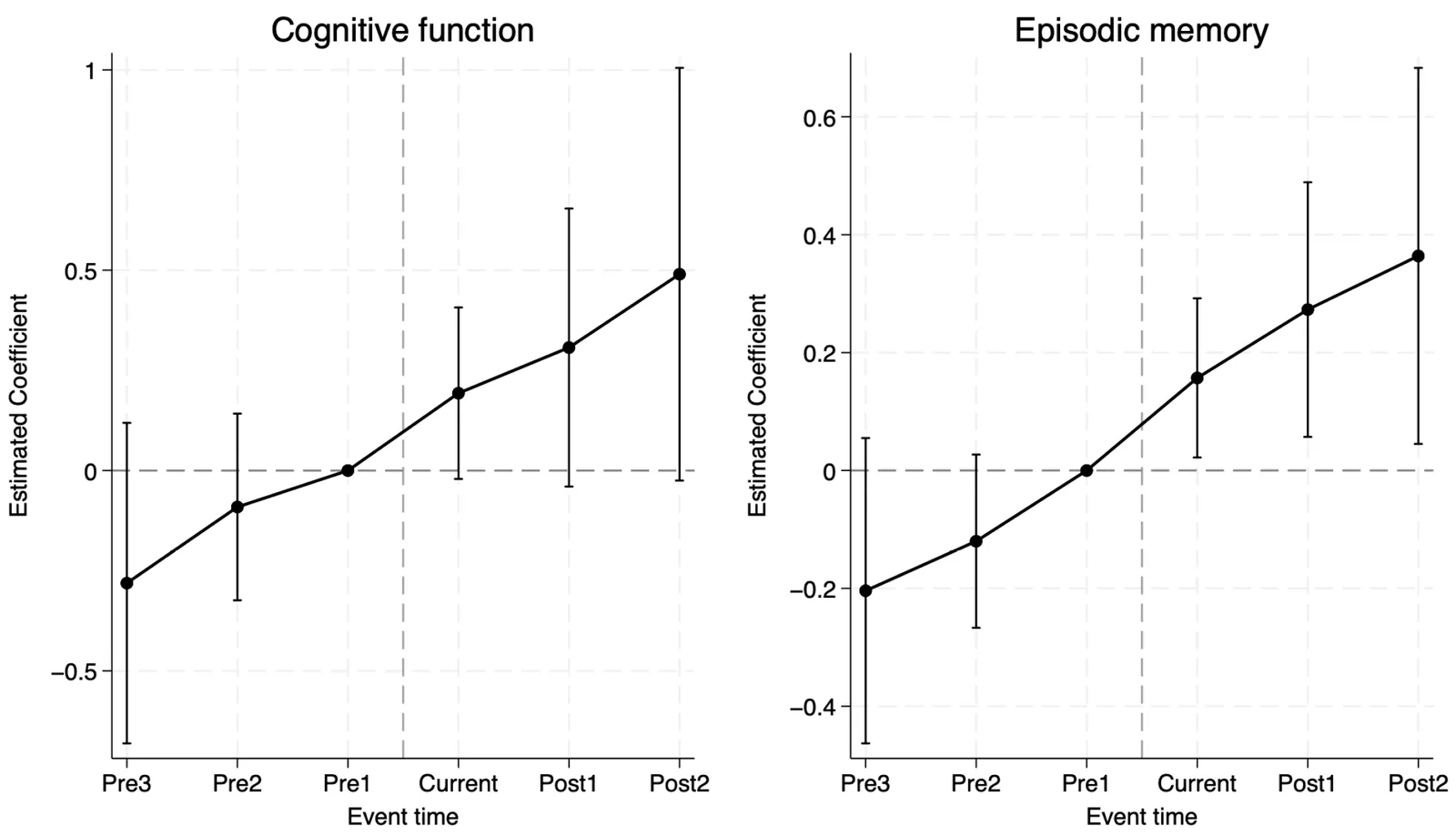
Dynamic effects of working beyond retirement on cognitive function and episodic memory. Note: Dots represent estimated coefficients, and error bars indicate 95% confidence intervals. The vertical dashed line indicates the time when working beyond retirement occurred, and the horizontal dashed line represents the zero-effect line.

To ensure the validity of the estimation results from the difference-in-differences model, it is necessary to conduct a parallel trend test, that is, to examine whether there were significant differences in the trends of cognitive function between the treatment and control groups before working beyond retirement occurred. If the two groups exhibited similar trends before working beyond retirement occurred, the parallel trend assumption can be considered satisfied. Table 4 and Figure 4 report the results of the parallel trend test based on the event study approach. Using the period immediately before the occurrence of working beyond retirement (Pre1) as the reference group, the estimated coefficients for the third period before the occurrence (Pre3), the second period before the occurrence (Pre2), the current period (Current), and the first and second periods after the occurrence (Post1 and Post2) are presented.

For cognitive function, before working beyond retirement occurred, the estimated coefficients for Pre3 (α = −0.281, *p* = 0.169) and Pre2 (α = −0.091, *p* = 0.443) were not significantly different from the reference period Pre1, indicating that the trends in cognitive function between the treatment and control groups did not significantly diverge before working beyond retirement occurred, thus satisfying the parallel trend assumption. In the current period (Current), the estimated coefficient was 0.193 and was significant at the 10% level (*p* = 0.078), suggesting that working beyond retirement had a positive effect on cognitive function in the year it occurred. The estimated coefficients for the first period after working beyond retirement occurred (Post1, α = 0.307, *p* = 0.082) and the second period after the intervention (Post2, α = 0.490, *p* = 0.063) were also significant at the 10% level, and the coefficients showed an increasing trend, indicating that the positive effect of working beyond retirement on overall cognitive function persisted over time.

For episodic memory, before working beyond retirement occurred, the estimated coefficients for Pre3 (α = −0.204, *p* = 0.122) and Pre2 (α = −0.120, *p* = 0.112) were not significantly different from the reference period Pre1, indicating that the trends in episodic memory between the treatment and control groups did not significantly diverge before working beyond retirement occurred, thus satisfying the parallel trend assumption. The estimated coefficient for the current period (Current) was 0.157 and was significant at the 5% level (*p* = 0.024), indicating that working beyond retirement had a significant positive effect on changes in episodic memory during the year it occurred. The estimated coefficients for the first period after occurrence (Post1, α = 0.273, *p* = 0.013) and the second period after occurrence (Post2, α = 0.364, *p* = 0.026) were also significant at the 5% level, and the coefficients continued to increase, further suggesting that the positive effect of working beyond retirement on episodic memory may exhibit some persistence and accumulation over time.

Taken together, the results do not reject the parallel trend assumption, providing support for the baseline regression results. From a stricter statistical perspective, the positive effect of working beyond retirement on cognitive function is mainly reflected in the dimension of episodic memory. Episodic memory reached the 5% significance level in both the current and subsequent periods after working beyond retirement occurred, with relatively robust estimates and continuously increasing coefficients. This finding further supports the dimension-specific patterns of the cognitive effects of working beyond retirement.

### 4.4. Robustness Checks

#### 4.4.1. Addressing Selection Bias

As shown by the baseline characteristics in Table 2, differences existed between the treatment and control groups in several variables, including educational attainment, residence, pension insurance coverage, and social participation. This study used nearest-neighbor matching with a caliper for propensity score matching to improve the comparability between the treatment and control groups. After matching, a total of 2390 observations were obtained, including 1744 observations in the treatment group and 646 observations in the control group. Subsequently, a balance test was conducted to assess the quality of the matching results. As shown in Table 5, after matching, the standardized bias of each variable was below 10%, and the post-matching *t*-tests failed to reject the null hypothesis at the 5% significance level, indicating that differences in major observable characteristics between the matched treatment and control groups were substantially reduced and that the matching results passed the balance test. Table 5 reports the balance test results for each variable before and after matching.

Based on propensity score matching (PSM), this study re-estimated the difference-in-differences (DID) model using the matched sample, thereby forming a PSM-DID estimation framework to test the robustness of the core results. Model (1) in Table 6 reports the estimation results based on the PSM-DID method. In terms of the core explanatory variable, after partially reducing selection bias, the effect pattern of working beyond retirement on cognitive function remained broadly consistent with the baseline regression results. Specifically, working beyond retirement still had a positive effect on overall cognitive function, although it was significant only at the 10% level (α = 0.253, *p* = 0.08). This change suggests that, after using propensity score matching to reduce the influence of sample selection bias, the estimated effect of working beyond retirement on overall cognitive function became more conservative. Some effects attributable to differences in observable characteristics were controlled, leading to a weakening of statistical significance. Nevertheless, the effect coefficient increased compared with the baseline regression coefficient of 0.198, suggesting that the positive relationship between working beyond retirement and improved overall cognitive function remained relatively stable after controlling for observable-variable bias.

From the perspective of specific cognitive dimensions, the coefficient of working beyond retirement on episodic memory was 0.329 and was significant at the 1% level (*p* < 0.001), representing an increase compared with the baseline regression coefficient of 0.158. This further indicates that the positive effect estimate for episodic memory remained stable in the matched sample and that episodic memory represents a key dimension affected by working beyond retirement. By contrast, the estimated coefficient for executive function was −0.075 and did not reach statistical significance, which is consistent with the baseline regression results and again indicates that the effect of working beyond retirement on executive function is relatively limited.

#### 4.4.2. Addressing Reverse Causality

To further improve the reliability of the estimation results, this study specifically addressed potential reverse causality within the PSM-DID framework. Specifically, individuals in the retirement-transition population with higher cognitive function may be more likely to obtain re-employment opportunities because of better memory and stronger executive function, and therefore may be included in the treatment group. By contrast, individuals with poorer cognitive function may be forced to choose full retirement because of insufficient labor capacity or employment competitiveness, and thus be included in the control group. If the treatment group already had cognitive advantages, the subsequently observed cognitive differences may have resulted from pre-existing differences rather than being fully attributable to the effect of working beyond retirement. To reduce the potential influence of reverse causality on the estimation results, this study adopted the following strategies. First, early-retirement individuals were excluded. On the basis of the age-based screening procedure, individuals who had experienced a non-working status before reaching the statutory retirement age were further excluded. Although these individuals met the age-transition criteria, they may have exited the labor market early due to poor health or other reasons, and their labor participation decisions may have been bidirectionally related to cognitive function. After this exclusion, the sample focused on normal retirement-transition individuals who made labor participation choices only after working until the statutory retirement age, thereby further reducing the possibility of reverse causality. Second, individuals with cognitive impairment at baseline were excluded. Because these individuals already had relatively low cognitive function, their subsequent decision to work beyond retirement may have been constrained by cognitive ability, and including them in the analysis could introduce reverse causality bias. After this exclusion, the remaining sample had a healthier baseline level of cognitive function, allowing for a further assessment of the robustness of the estimation results. The specific procedure followed previous research (Luo et al., 2024): individuals whose baseline cognitive function scores were at least one standard deviation below the mean of their age group were defined as having mild cognitive impairment and were excluded.

Table 6 reports the relevant estimation results. Model (2) presents the results after excluding early-retirement samples, and Model (3) presents the results after excluding individuals with mild cognitive impairment at baseline.

According to the estimation results of Model (2), after excluding early-retirement samples, the coefficient of working beyond retirement on overall cognitive function was 0.269 and was significant at the 10% level; the coefficient for episodic memory was 0.345 and was significant at the 1% level; and the coefficient for executive function was −0.077 and did not reach statistical significance. Compared with the baseline regression, the significance level of overall cognitive function in Model (2) decreased, but the effect coefficient remained positive and marginally significant.

According to the estimation results of Model (3), after excluding individuals with mild cognitive impairment at baseline, the coefficient of working beyond retirement on overall cognitive function was 0.048 and did not reach statistical significance; the coefficient for episodic memory was 0.244 and was significant at the 1% level; and the coefficient for executive function was −0.196 and did not reach statistical significance.

These results further confirm that the cognitive effects of working beyond retirement are dimension-specific, with episodic memory showing relatively more stable estimates. The change in the significance of overall cognitive function suggests that when the sample is restricted to individuals with higher baseline cognitive function, the marginal improvement of working beyond retirement on overall cognition is limited, but the positive relationship between working beyond retirement and improved episodic memory remains.

### 4.5. Heterogeneity Analysis

The baseline regression results show that working beyond retirement has a significant effect on cognitive function among the retirement-transition population, and this effect is mainly reflected in the dimension of episodic memory. However, existing studies suggest that the health effects of working beyond retirement may vary across different groups, and the direction and intensity of these effects may be moderated by individual characteristics, health status, behavioral habits, and specific behavioral patterns. Focusing only on the average treatment effect may obscure these differences and hinder a deeper understanding of the underlying mechanisms and boundary conditions through which working beyond retirement operates. Based on this, this study further conducted heterogeneity analyses from multiple dimensions, including demographic characteristics and health status, to more comprehensively reveal group differences in the cognitive effects of working beyond retirement. Since the effect of working beyond retirement on executive function was not significant in either the baseline regression or the robustness checks, executive function was not included as an outcome variable in this section.

#### 4.5.1. Heterogeneity Analysis by Demographic Characteristics

To further reveal group heterogeneity in the cognitive effects of working beyond retirement, this study conducted subgroup regressions from the perspective of demographic characteristics. Table 7 reports the relevant estimation results.

From the perspective of gender, the effect of working beyond retirement on cognitive function differs markedly. In the male sample, the coefficient of working beyond retirement on overall cognitive function was 0.352 and was significant at the 1% level; the coefficient for episodic memory was 0.501 and was also significant at the 1% level. In the female sample, the coefficient for overall cognitive function was 0.106 and did not reach statistical significance; the coefficient for episodic memory was 0.234 and was significant only at the 10% level. Compared with the baseline regression results, in which overall cognitive function was significant at the 5% level and episodic memory was significant at the 1% level, the positive cognitive effects among men were more pronounced than the average effect, whereas the effects among women were mainly reflected in episodic memory and showed weaker statistical significance. This indicates that the cognitive effects of working beyond retirement are more pronounced among men.

From the perspective of residence, both urban and rural residents experienced positive cognitive effects from working beyond retirement, but the levels of statistical significance differ. In the urban sample, the coefficient of working beyond retirement on overall cognitive function was 0.155 and did not reach statistical significance; the coefficient for episodic memory was 0.329 and was significant at the 1% level. In the rural sample, the coefficient for overall cognitive function was 0.181 and did not reach statistical significance; the coefficient for episodic memory was 0.268 and was significant at the 10% level. Compared with the baseline regression result in which episodic memory was significant at the 1% level, the effect on episodic memory remained highly significant in the urban sample, whereas its statistical significance was weaker in the rural sample. This suggests that the positive effect of working beyond retirement on episodic memory is more robust among urban residents.

#### 4.5.2. Heterogeneity Analysis by Health Status

Individual health status may influence the magnitude of cognitive gains from working beyond retirement. Therefore, this study conducted subgroup regressions from three dimensions: chronic disease, depressive symptoms, and self-rated health. A depressive symptoms score of 10 or above was defined as having depressive symptoms (Luo et al., 2018). Table 8 reports the relevant estimation results.

From the perspective of chronic disease, the positive effect of working beyond retirement on episodic memory was observed regardless of whether individuals had chronic disease. Specifically, in the sample without chronic disease, the coefficient of working beyond retirement on episodic memory was 0.408 and was significant at the 5% level; in the sample with chronic disease, the coefficient for episodic memory was 0.272 and was also significant at the 5% level. In terms of coefficient size, the estimated effect among individuals without chronic disease (0.408) was greater than that among individuals with chronic disease (0.272), indicating that retirement-transition individuals without chronic disease showed a stronger positive effect of working beyond retirement on episodic memory. In the two samples, the coefficients of working beyond retirement on overall cognitive function were 0.217 and 0.283, respectively, neither of which reached statistical significance.

From the perspective of depressive symptoms, the effect of working beyond retirement on cognitive function differed markedly. In the sample without depressive symptoms, the coefficient of working beyond retirement on episodic memory was 0.348 and was significant at the 1% level; in the sample with depressive symptoms, the coefficient for episodic memory was 0.231 and did not reach statistical significance. In terms of coefficient size, the estimated effect among individuals without depressive symptoms (0.348) was greater than that among individuals with depressive symptoms (0.231), and the former showed stronger statistical significance. This indicates that the positive effect of working beyond retirement on episodic memory was mainly observed among individuals without depressive symptoms.

From the perspective of self-rated health, in the sample with fair or better self-rated health, the coefficient of working beyond retirement on episodic memory was 0.155 and was significant at the 5% level; in the sample with below-fair self-rated health, the coefficient for episodic memory was 0.159 and did not reach statistical significance. The coefficient sizes were broadly similar between the two groups, but only the group with better self-rated health reached statistical significance. This suggests that the positive effect of working beyond retirement on episodic memory is more robust among individuals with better self-rated health.

In summary, the heterogeneity analysis by health status shows that the positive effect of working beyond retirement on episodic memory is more pronounced among individuals without chronic disease, without depressive symptoms, and with better self-rated health, suggesting that good health status is an important condition for obtaining greater cognitive gains from working beyond retirement.

#### 4.5.3. Heterogeneity Analysis by Work-Type Change

To further examine whether work-type change influences the cognitive effects of working beyond retirement, this study conducted the analysis within the PSM-DID framework. Table 9 reports the estimation results.

Overall, both the group with work-type change and the group without work-type change showed positive effects of working beyond retirement on episodic memory. In the group without work-type change, the coefficient of working beyond retirement on episodic memory was 0.319 and was significant at the 1% level, while the coefficient for overall cognitive function was 0.272 and did not reach statistical significance. In the group with work-type change, the coefficient of working beyond retirement on episodic memory was 0.413 and was significant at the 1% level, while the coefficient for overall cognitive function was 0.304 and also did not reach statistical significance. In terms of coefficient size, the benefit for episodic memory in the group with work-type change may represent an important contextual factor influencing the magnitude of cognitive effects from working beyond retirement.

Among different types of work-type transitions, the agricultural-to-non-agricultural transition group showed the most prominent cognitive benefits. The coefficient of working beyond retirement on overall cognitive function was 0.683 and was significant at the 5% level, while the coefficient for episodic memory was 0.487 and was significant at the 1% level. By contrast, in the non-agricultural-to-agricultural transition group, the coefficients of working beyond retirement on both episodic memory and overall cognitive function did not reach statistical significance, indicating that this transition pathway did not produce significant cognitive benefits.

In summary, the heterogeneity analysis by work-type change suggests that work-type change may strengthen the cognitive effects of working beyond retirement, but it is not a necessary condition for observing cognitive benefits. Among different transition pathways, the agricultural-to-non-agricultural transition exhibited more pronounced cognitive benefits; therefore, these findings provide partial support for Hypothesis 2.

## 5. Discussion

This study found that working beyond retirement had a positive effect on cognitive function among the retirement-transition population. This finding is consistent with the cognitive reserve theory. According to the cognitive reserve theory, continued occupational engagement may help individuals maintain cognitive capacity by providing cognitive stimulation, social interactions, and opportunities for role maintenance. Previous studies have also shown that occupational activities and social participation are important sources of cognitive reserve accumulation in later life (Livingston et al., 2024; Kuiper et al., 2016). Therefore, this study extends previous research by suggesting that post-retirement work participation may have not only economic value but also potential cognitive health value. However, because the decision to work beyond retirement may still be influenced by unobserved individual factors, such as baseline health status, functional ability, and socioeconomic conditions, the findings should be interpreted cautiously. Although this study employed a staggered difference-in-differences approach and conducted multiple robustness checks, further validation under more rigorous causal identification frameworks is still needed. Further analyses showed that the effect of working beyond retirement was mainly observed in the episodic memory dimension, whereas no significant effect was found for executive function. This dimensional difference may be related to variations in the sensitivity of different cognitive functions to external stimulation and cognitive challenges. This difference across cognitive dimensions may reflect variations in information processing patterns and everyday utilization contexts between different cognitive functions. Episodic memory involves the retrieval of past events and their associated contextual information (Eichenbaum et al., 2007; Davachi et al., 2003). Continuing to work after retirement often involves maintaining relatively structured daily routines and ongoing exposure to people, events, and information in the workplace, which may increase the everyday use of relevant memory processes. Previous research has shown that sustained participation in activities requiring new skill acquisition and cognitive engagement can improve episodic memory performance among older adults (D. C. Park et al., 2014). In contrast, executive function involves more complex cognitive processes, including goal maintenance, task organization, and behavioral adjustment according to changing demands (Duncan, 2010). General labor participation may not provide equivalent opportunities to exercise these abilities. Therefore, the cognitive effects of working beyond retirement may be more likely to manifest in episodic memory, but may not produce comparable effects on executive function. It should be noted that the above explanation based on cognitive reserve theory is primarily a theoretical interpretation. This study did not directly examine cognitive stimulation, social interaction, or other potential mediating pathways; therefore, cognitive reserve should not be regarded as the sole mechanism underlying the observed effects. Beyond pathways related to cognitive reserve, continued work may also influence cognitive function through other pathways, including increased income, improved psychological well-being, expanded social networks, and more active lifestyles (Takase et al., 2024; Kim & Park, 2023). These potential mechanisms require further investigation in future studies. Meanwhile, continued work does not necessarily generate equivalent cognitive gains for all individuals, as the quality of work participation and working conditions may influence its effects on health outcomes (Fattori et al., 2024). Compared with jobs characterized by greater autonomy, social interaction, and opportunities for skill utilization, low-quality or highly demanding work may provide insufficient cognitive stimulation and may even increase psychological and physical burdens (Aichele et al., 2024).

This study finds that the cognitive effects of working beyond retirement vary by individual characteristics. In terms of demographic characteristics, cognitive benefits are more pronounced among men. One possible explanation is that men have traditionally relied more on occupational activities to establish social identity and social connections. Continuing to work after retirement may help them maintain existing occupational roles and social networks (Cohn-Schwartz & Naegele, 2024). Compared with men, women generally undertake more family caregiving responsibilities (Sharma et al., 2016; Zhang & Feng, 2024), their willingness and opportunities to continue working after retirement may also be jointly affected by gender roles, health status, and socioeconomic characteristics (Zaccagni et al., 2024). Even when they choose to continue working, family responsibilities may make it difficult for them to balance work and life, increase stress, and thereby attenuate the cognitive benefits of working beyond retirement. In addition, cognitive benefits among both urban and rural groups are mainly reflected in episodic memory, with the effect being more evident and robust in the urban sample. A possible explanation is that post-retirement work participation among urban residents often has stronger social participation characteristics. Continuing to work may not only provide income but also help maintain occupational identity, expand social interactions, and offer richer cognitive stimulation (Lu et al., 2025). By contrast, because China’s pension policies started relatively late, many farmers have difficulty relying on pensions for their livelihoods (Ministry of Human Resources and Social Security of China, 2023; National Bureau of Statistics of China, 2022). Most rural residents are forced to choose survival-driven working beyond retirement to maintain their livelihoods, placing greater emphasis on economic returns rather than the value of work itself. Out of a strong sense of responsibility toward their children and concerns about future economic risks, they often choose to continue working throughout life rather than engage in a reasonable form of working beyond retirement (C. Li et al., 2024; Tan et al., 2022), and such high-intensity work may have negative effects on health. In terms of health status, individuals without chronic disease, without depressive symptoms, and with better self-rated health benefit significantly in the dimension of episodic memory, whereas those with these health problems obtain relatively limited cognitive benefits. This difference may be explained by the influence of health status on the quality of work participation and the level of cognitive stimulation obtained from work activities. Individuals with better health foundations generally have stronger functional capacity, allowing them to participate in work activities more consistently and obtain more positive experiences from work (Tur-Sinai et al., 2024), thereby allowing them to derive greater potential cognitive benefits from working beyond retirement. Conversely, chronic diseases and depressive symptoms may restrict individuals’ ability to participate in work, reduce work engagement and social interactions (Shiri et al., 2025), making continued work less likely to translate into effective cognitive stimulation. Therefore, baseline health status may represent an important boundary condition influencing the cognitive effects of working beyond retirement. From the perspective of behavioral context, changes in work type may influence the magnitude of the cognitive effects of working beyond retirement. According to continuity theory, adaptation in later life involves both maintaining existing lifestyles and social roles and adapting to environmental changes within certain limits. Therefore, continuing previous work after retirement may support cognitive functioning by maintaining occupational identity, daily routines, and social connections. In contrast, work-type change may introduce new task demands and social environments while maintaining certain aspects of continuity, thereby increasing cognitive engagement during the adaptation process (Coleman et al., 2023). From this perspective, an agricultural-to-non-agricultural transition may represent a more substantial change in the occupational environment. During this transition, individuals enter more diverse work environments and need to adapt to new job tasks, skill requirements, and social networks, which may increase cognitive stimulation and help maintain cognitive flexibility (Kobayashi et al., 2024). In contrast, the transition from non-agricultural to agricultural work may involve more repetitive tasks and reduced job complexity, making it less likely to generate comparable cognitive stimulation or provide equivalent cognitive challenges (Lenehan et al., 2023). Therefore, significant cognitive benefits were not observed in this transition pathway.

Based on these findings, this study provides several practical implications. First, working beyond retirement should be incorporated into the health promotion system, and the objectives of later-life employment policies should be reconstructed from the perspective of health benefits. Current policies mainly focus on the economic value of delayed retirement in terms of pension sustainability and labor supply, while insufficient attention has been paid to the potential cognitive health value of working beyond retirement. It is recommended that a “health benefit assessment of later-life employment” module be added to the existing policy evaluation framework. Health dimensions such as improvements in cognitive function, maintenance of psychological well-being, and enhancement of quality of life should be incorporated into policy evaluation systems, preventing policy assessments from being limited solely to economic indicators such as employment rates and pension replacement rates (Zacher et al., 2021). Second, rural older adults should be guided from “survival-oriented labor” toward “value-oriented participation.” Basic pension protection for urban and rural residents should be further improved to create a rights-based space for rural older adults to withdraw from work when needed (Jiang, 2026). At the same time, localized, low-intensity, and organized age-friendly employment opportunities should be developed to provide the rural retirement-transition population with more channels for social participation, allowing working beyond retirement to shift from a survival behavior driven by livelihood pressure to a positive choice with social participation value. Third, job quality evaluation and health-matching mechanisms should be established to promote the quality upgrading of later-life employment. Job quality evaluation standards for older adults should be developed. Drawing on age-friendly workplace measurement tools, age-friendly jobs should be evaluated across dimensions including workplace adaptability, career development opportunities, health protection, skill utilization, and inclusiveness (Eppler-Hattab et al., 2020). Meanwhile, a later-life employment service system integrating “health screening–classified guidance–precise matching” should be established. Relying on grassroots health services and employment service platforms, health assessments should be conducted among older adults who intend to continue working. Suitable jobs should then be matched according to their health status, work ability, and employment preferences, thereby enabling working beyond retirement to become a facilitator of cognitive health.

This study has the following limitations, which need to be further addressed in future research. First, endogeneity remains a concern. Due to the lack of targeted survey data, although this study used a staggered difference-in-differences approach and conducted multiple robustness checks, individuals’ decisions to work beyond retirement may still be influenced by unobserved factors, making it difficult to completely eliminate endogeneity. Future research may use more rigorous causal inference methods, such as instrumental variable approaches and regression discontinuity designs, for further verification. Second, the measurement dimensions of cognitive function are limited. This study mainly focused on the two dimensions of episodic memory and executive function and did not fully cover other cognitive domains, such as language ability and processing speed. Future research may introduce more comprehensive neuropsychological assessment tools to further expand the research dimensions. Third, the behavioral characteristics of working beyond retirement require further refinement. Due to data limitations, this study defined working beyond retirement primarily based on labor participation status observed in each CHARLS survey wave and was unable to capture short-term exits, temporary interruptions, or re-employment processes occurring between survey waves. In addition, this study was unable to further distinguish characteristics such as work intensity, working hours, and work motivation. Future studies could incorporate higher-frequency longitudinal data to further characterize the dynamic processes of working beyond retirement and examine the cognitive effects of different patterns of labor participation.

## 6. Conclusions

This study used data from the 2013–2020 waves of the China Health and Retirement Longitudinal Study (CHARLS) and employed a staggered difference-in-differences approach to examine the effect of working beyond retirement on cognitive function among the retirement-transition population. The findings showed that working beyond retirement had a positive effect on cognitive function, but this effect was mainly observed in the episodic memory dimension, while its influence on executive function was limited. The core findings remained consistent after multiple robustness checks. Further analyses indicated that the cognitive effects of working beyond retirement varied across population groups. Men, urban residents, and individuals with better health status showed more pronounced cognitive benefits. Changes in work type may influence the magnitude of these effects, and transitions from agricultural to non-agricultural work may generate greater cognitive benefits. Overall, “working beyond retirement” represents not only a continuation of employment after retirement but also a form of social participation with potential cognitive health benefits in the context of active aging. These findings suggest that flexible retirement and older adult employment policies may appropriately consider the potential health benefits associated with continued labor participation. However, the magnitude and applicability of its effects remain constrained by factors such as individual health status, occupational type, and working conditions. Therefore, these findings should not be generalized directly to all retired populations. Future research should incorporate richer data sources and more rigorous research designs to further investigate how different patterns of labor participation and their underlying mechanisms influence cognitive health in later life.

## Figures and Tables

**Table 1:** Variable Definitions and Coding.

Variable Category	Variable Name	Coding
Independent variable	Working beyond retirement	1 = Yes; 0 = No
Outcome variable	Cognitive function	Sum of episodic memory and executive function scores
	Episodic memory	Higher values indicate better episodic memory
	Executive function	Higher values indicate better executive function
Control variable	Age	0 = 45–54 years; 1 = 55–59 years; 2 = 60–67 years
	Gender	0 = Female; 1 = Male
	Educational attainment	0 = Below primary school; 1 = Primary school; 2 = Middle school; 3 = High school or above
	Marital status	0 = Not partnered; 1 = Partnered
	Residence	0 = Urban; 1 = Rural
	Pension insurance	0 = No; 1 = Yes
	Medical insurance	0 = No; 1 = Yes
	Drinking status	0 = No; 1 = Yes
	Smoking status	0 = No; 1 = Yes
	Chronic disease	0 = No; 1 = Yes
	Self-rated health	0 = Very poor; 1 = Poor; 2 = Fair; 3 = Good; 4 = Very good
	Depressive symptoms	Higher values indicate more severe depressive symptoms
	Bodily pain	0 = No; 1 = Yes
	Social participation	0 = No; 1 = Yes

**Table 2:** Descriptive Statistics of Variables.

Variable	Full Sample (*n* = 1336)	Treatment Group (*n* = 1122)	Control Group (*n* = 214)
**Age**	52.103 (4.931)	52.120 (4.956)	52.014 (4.812)
**Gender**			
Female	654 (48.95)	555 (49.47)	99 (46.26)
Male	682 (51.05)	567 (50.53)	115 (53.74)
**Educational attainment**			
Below primary school	213 (15.94)	199 (17.74)	14 (6.54)
Primary school	424 (31.74)	362 (32.26)	62 (28.97)
Middle school	427 (31.96)	353 (31.46)	74 (34.58)
High school or above	272 (20.36)	208 (18.54)	64 (29.91)
**Marital status**			
Not partnered	45 (3.37)	32 (2.85)	13 (6.07)
Partnered	1291 (96.63)	1090 (97.15)	201 (93.93)
**Residence**			
Urban	559 (41.84)	407 (36.27)	152 (71.03)
Rural	777 (58.16)	715 (63.73)	62 (28.97)
**Pension insurance**			
No	1279 (95.73)	1094 (97.50)	185 (86.45)
Yes	57 (4.27)	28 (2.50)	29 (13.55)
**Medical insurance**			
No	47 (3.52)	40 (3.57)	7 (3.27)
Yes	1289 (96.48)	1082 (96.43)	207 (96.73)
**Chronic disease**			
No	471 (35.25)	398 (35.47)	73 (34.11)
Yes	865 (64.75)	724 (64.53)	141 (65.89)
**Depressive symptoms**	6.769 ± 5.209	6.773 ± 5.193	6.752 ± 5.302
**Drinking status**			
No	791 (59.21)	672 (59.89)	119 (55.61)
Yes	545 (40.79)	450 (40.11)	95 (44.39)
**Smoking status**			
No	966 (72.31)	817 (72.82)	149 (69.63)
Yes	370 (27.69)	305 (27.18)	65 (30.37)
**Self-rated health**			
Very poor	23 (1.72)	19 (1.69)	4 (1.87)
Poor	175 (13.10)	151 (13.46)	24 (11.21)
Fair	740 (55.39)	615 (54.81)	125 (58.41)
Good	234 (17.51)	196 (17.47)	38 (17.76)
Very good	164 (12.28)	141 (12.57)	23 (10.75)
**Social participation**			
No	757 (56.66)	647 (57.66)	110 (51.40)
Yes	579 (43.34)	475 (42.34)	104 (48.60)

**Table 3:** Effect of Working Beyond Retirement on Cognitive Function under the Difference-in-Differences Framework.

Variable	Cognitive Function			Episodic Memory			Executive Function		
	*α* [95% CI]	*SE*	*p*	*α* [95% CI]	*SE*	*p*	*α* [95% CI]	*SE*	*p*
Working beyond retirement	0.198 [0.018, 0.378]	0.092	0.031	0.158 [0.046, 0.270]	0.057	0.006	0.039 [−0.090, 0.168]	0.066	0.553
**Age**									
45–54 years	Ref.	Ref.	Ref.	Ref.	Ref.	Ref.	Ref.	Ref.	Ref.
55–59 years	−0.139 [−0.425, 0.147]	0.146	0.341	−0.164 [−0.350, 0.022]	0.095	0.084	0.024 [−0.180, 0.228]	0.104	0.814
60 years and above	−0.761 [−1.159, −0.363]	0.203	<0.001	−0.614 [−0.875, −0.353]	0.133	<0.001	−0.148 [−0.426, 0.130]	0.142	0.300
**Educational attainment**									
Below primary school	Ref.	Ref.	Ref.	Ref.	Ref.	Ref.	Ref.	Ref.	Ref.
Primary school	0.317 [−0.120, 0.754]	0.223	0.156	0.234 [−0.031, 0.499]	0.135	0.084	0.084 [−0.247, 0.415]	0.169	0.620
Middle school	−0.074 [−0.607, 0.459]	0.272	0.787	0.048 [−0.287, 0.383]	0.171	0.779	−0.122 [−0.502, 0.258]	0.194	0.531
High school or above	0.107 [−0.601, 0.815]	0.361	0.767	−0.053 [−0.521, 0.415]	0.239	0.824	0.160 [0.013, 0.307]	0.075	0.033
**Marital status**									
Not partnered	Ref.	Ref.	Ref.	Ref.	Ref.	Ref.	Ref.	Ref.	Ref.
Partnered	−0.266 [−0.879, 0.347]	0.313	0.396	−0.178 [−0.556, 0.200]	0.193	0.355	−0.088 [−0.562, 0.386]	0.242	0.718
**Pension insurance**									
No	Ref.	Ref.	Ref.	Ref.	Ref.	Ref.	Ref.	Ref.	Ref.
Yes	0.038 [−0.154, 0.230]	0.098	0.696	0.070 [−0.057, 0.197]	0.065	0.281	−0.032 [−0.173, 0.109]	0.072	0.657
**Medical insurance**									
No	Ref.	Ref.	Ref.	Ref.	Ref.	Ref.	Ref.	Ref.	Ref.
Yes	−0.120 [−0.596, 0.356]	0.243	0.621	−0.040 [−0.318, 0.238]	0.142	0.776	−0.080 [−0.417, 0.257]	0.172	0.642
**Chronic disease**									
No	Ref.	Ref.	Ref.	Ref.	Ref.	Ref.	Ref.	Ref.	Ref.
Yes	−0.175 [−0.391, 0.041]	0.110	0.113	0.077 [−0.072, 0.226]	0.076	0.313	−0.251 [−0.404, −0.098]	0.078	0.001
**Depressive symptoms**	−0.042 [−0.060, −0.024]	0.009	<0.001	−0.015 [−0.025, −0.005]	0.005	0.004	−0.027 [−0.039, −0.015]	0.006	<0.001
**Drinking status**									
No	Ref.	Ref.	Ref.	Ref.	Ref.	Ref.	Ref.	Ref.	Ref.
Yes	0.266 [0.062, 0.470]	0.104	0.010	0.099 [−0.026, 0.224]	0.064	0.120	0.167 [0.022, 0.312]	0.074	0.024
Smoking status									
No	Ref.	Ref.	Ref.	Ref.	Ref.	Ref.	Ref.	Ref.	Ref.
Yes	−0.141 [−0.417, 0.135]	0.141	0.317	−0.048 [−0.219, 0.123]	0.087	0.579	−0.093 [−0.305, 0.119]	0.108	0.387
**Self-rated health**									
Very poor	Ref.	Ref.	Ref.	Ref.	Ref.	Ref.	Ref.	Ref.	Ref.
Poor	0.158 [−0.299, 0.615]	0.233	0.497	0.132 [−0.162, 0.426]	0.150	0.379	0.026 [−0.305, 0.357]	0.169	0.878
Fair	0.271 [−0.194, 0.736]	0.237	0.255	0.165 [−0.131, 0.461]	0.151	0.275	0.105 [−0.232, 0.442]	0.172	0.542
Good	0.361 [−0.127, 0.849]	0.249	0.147	0.180 [−0.138, 0.498]	0.162	0.266	0.181 [−0.172, 0.534]	0.180	0.316
Very good	0.165 [−0.347, 0.677]	0.261	0.527	0.098 [−0.239, 0.435]	0.172	0.569	0.067 [−0.303, 0.437]	0.189	0.723
**Social participation**									
No	Ref.	Ref.	Ref.	Ref.	Ref.	Ref.	Ref.	Ref.	Ref.
Yes	0.152 [0.001, 0.303]	0.077	0.049	0.094 [−0.002, 0.190]	0.049	0.055	0.058 [−0.050, 0.166]	0.055	0.290
N	4918			4918			4918		

Note: Standard errors are robust standard errors clustered at the individual level.

**Table 4:** Parallel Trend Test Results.

Variable	Cognitive Function			Episodic Memory		
	α [95% CI]	*SE*	*p*	α [95% CI]	*SE*	*p*
Pre3	−0.281 [−0.681, 0.119]	0.204	0.169	−0.204 [−0.463, 0.055]	0.132	0.122
Pre2	−0.091 [−0.324, 0.142]	0.119	0.443	−0.120 [−0.267, 0.027]	0.075	0.112
Pre1	Ref.	Ref.	Ref.	Ref.	Ref.	Ref.
Current	0.193 [−0.021, 0.407]	0.109	0.078	0.157 [0.022, 0.292]	0.069	0.024
Post1	0.307 [−0.040, 0.654]	0.177	0.082	0.273 [0.057, 0.489]	0.110	0.013
Post2	0.490 [−0.025, 1.005]	0.263	0.063	0.364 [0.045, 0.683]	0.163	0.026

Note: Standard errors are robust standard errors clustered at the individual level. Since the baseline regression results showed that the effect of working beyond retirement on executive function was not statistically significant, executive function was not included as an outcome variable in this section.

**Table 5:** Balance Test.

Variable	Sample Matching	Mean		Standardized Bias (%)	Bias Reduction (%)	*t*-Test	
		Treatment Group	Control Group			*t*	*p > |t|*
Educational attainment	U	2.533	2.905	−38.7		−9.50	<0.001
	M	2.600	2.600	0.0	100.0	−0.20	0.845
Gender	U	0.517	0.553	−7.3		−1.83	0.067
	M	0.460	0.454	1.1	84.4	0.37	0.710
Marital status	U	0.955	0.941	6.5		1.73	0.085
	M	0.974	0.981	−3.6	44.6	−1.73	0.085
Residence	U	0.644	0.293	75.1		18.56	<0.001
	M	0.609	0.596	2.7	96.5	0.58	0.565
Pension insurance	U	0.401	0.519	−23.9		−6.06	<0.001
	M	0.362	0.367	−1.1	95.6	−0.35	0.724
Smoking status	U	0.277	0.318	−8.9		−2.28	0.023
	M	0.243	0.237	1.3	85.1	0.47	0.642
Depressive symptoms	U	7.307	6.803	8.9		2.21	0.027
	M	6.565	6.680	−2.0	77.2	−0.68	0.500
Social participation	U	0.472	0.518	−9.3		−2.33	0.020
	M	0.496	0.503	−1.4	84.4	−0.47	0.640

**Table 6:** Regression Results of Robustness Checks.

Variable	(1) Propensity Score Matching		(2) Excluding Early-Retirement Samples		(3) Excluding Individuals with Mild Cognitive Impairment at Baseline	
	α [95% CI]	*SE*	α	*SE*	α [95% CI]	*SE*
Cognitive function	0.253 * [−0.029, 0.535]	0.144	0.269 * [−0.025, 0.563]	0.150	0.048 [−0.328, 0.424]	0.192
Episodic memory	0.329 *** [0.149, 0.509]	0.092	0.345 *** [0.159, 0.531]	0.095	0.244 *** [0.060, 0.428]	0.094
Executive function	−0.075 [−0.281, 0.131]	0.105	−0.077 [−0.171, 0.017]	0.048	−0.196 [−0.447, 0.055]	0.128
N	2390		2166		2087	

Note: Standard errors are robust standard errors clustered at the individual level. *** and * indicate statistical significance at the 1% and 10% levels, respectively.

**Table 7:** Results of the Heterogeneity Analysis by Demographic Characteristics.

Variable	(1) Gender		(2) Residence	
	Male	Female	Urban	Rural
Cognitive function	0.352 *** [0.117, 0.587]	0.106 [−0.072, 0.284]	0.155 [−0.029, 0.339]	0.181 [−0.031, 0.393]
Episodic memory	0.501 *** [0.197, 0.805]	0.234 * [−0.021, 0.489]	0.329 *** [0.110, 0.548]	0.268 * [−0.026, 0.562]
N	1122	1268	1249	1141

Note: Values in brackets represent 95% confidence intervals. Standard errors are robust standard errors clustered at the individual level. *** and * indicate statistical significance at the 1% and 10% levels, respectively.

**Table 8:** Results of the Heterogeneity Analysis by Health Status.

Variable	(1) Chronic Disease		(2) Depressive Symptoms		(3) Self-Rated Health	
	No	Yes	No	Yes	Below Fair	Fair or Above
Cognitive function	0.217 [−0.086, 0.520]	0.283 [−0.073, 0.639]	0.095 [−0.108, 0.298]	0.620 [−0.042, 1.282]	0.381 [−0.041, 0.803]	0.156 [−0.012, 0.324]
Episodic memory	0.408 ** [0.062, 0.754]	0.272 ** [0.056, 0.488]	0.348 *** [0.154, 0.542]	0.231 [−0.078, 0.540]	0.159 [−0.105, 0.423]	0.155 ** [0.018, 0.292]
N	672	1718	1843	547	843	4075

Note: Values in brackets represent 95% confidence intervals. Standard errors are robust standard errors clustered at the individual level. *** and ** indicate statistical significance at the 1% and 5% levels, respectively. Model 3 used the baseline DID model because the number of matched individuals with below-fair self-rated health was relatively small.

**Table 9:** Results of the Heterogeneity Analysis by Work-Type Change.

Variable	Work-Type Change: No	Work-Type Change: Yes	Agricultural-to-Non-Agricultural Transition	Non-Agricultural-to-Agricultural Transition
Cognitive function	0.272 [−0.012, 0.556]	0.304 [−0.015, 0.623]	0.683 ** [0.147, 1.219]	−0.130 [−0.491, 0.231]
Episodic memory	0.319 *** [0.140, 0.498]	0.413 *** [0.206, 0.620]	0.487 *** [0.216, 0.758]	0.146 [−0.096, 0.388]
N	2190	1893	896	997

Note: Values in brackets represent 95% confidence intervals. Standard errors are robust standard errors clustered at the individual level. *** and ** indicate statistical significance at the 1% and 5% levels, respectively.

## Data Availability

The data analyzed in this study are publicly available from the China Health and Retirement Longitudinal Study (CHARLS). The original CHARLS datasets can be accessed by registered users upon application through the CHARLS official data platform (https://charls.charlsdata.com/, accessed on 22 October 2025). The processed analytic dataset constructed for the current study is available from the corresponding author upon reasonable request.

## Abbreviations

CHARLS: China Health and Retirement Longitudinal Study; DID: difference-in-differences; MCI: mild cognitive impairment; PSM: propensity score matching; PSM-DID: propensity score matching combined with difference-in-differences; TICS: Telephone Interview for Cognitive Status.

## References

[B1-behavsci-16-01527] Aichele S., Payton A., Robinson A. C., Rabbitt P. (2024). Occupation-related differences in cognitive aging: Comparative effects of job type, skill level, and education. Intelligence.

[B2-behavsci-16-01527] Atchley R. C. (1989). A continuity theory of normal aging. The Gerontologist.

[B3-behavsci-16-01527] Atchley R. C. (1999). Continuity and adaptation in aging: Creating positive experiences.

[B4-behavsci-16-01527] Bertogg A., Leist A. K. (2023). Gendered life courses and cognitive functioning in later life: The role of context-specific gender norms and lifetime employment. European Journal of Ageing.

[B5-behavsci-16-01527] Celidoni M., Dal Bianco C., Weber G. (2017). Retirement and cognitive decline: A longitudinal analysis using SHARE data. Journal of Health Economics.

[B6-behavsci-16-01527] Cheng J. (2014). “Working after retirement”: A typical phenomenon in transitional China. Labor Economics Research.

[B7-behavsci-16-01527] Cohn-Schwartz E., Naegele L. (2024). Employment over the life course and post-retirement social networks: A gendered perspective. International Psychogeriatrics.

[B8-behavsci-16-01527] Coleman M. E., Roessler M. E. H., Peng S., Roth A. R., Risacher S. L., Saykin A. J., Apostolova L. G., Perry B. L. (2023). Social enrichment on the job: Complex work with people improves episodic memory, promotes brain reserve, and reduces the risk of dementia. Alzheimer’s & Dementia.

[B9-behavsci-16-01527] Dahan L., Rampon C., Florian C. (2020). Age-related memory decline, dysfunction of the hippocampus and therapeutic opportunities. Progress in Neuro-Psychopharmacology and Biological Psychiatry.

[B10-behavsci-16-01527] Dajani D. R., Uddin L. Q. (2015). Demystifying cognitive flexibility: Implications for clinical and developmental neuroscience. Trends in Neurosciences.

[B11-behavsci-16-01527] Davachi L., Mitchell J. P., Wagner A. D. (2003). Multiple routes to memory: Distinct medial temporal lobe processes build item and source memories. Proceedings of the National Academy of Sciences of the United States of America.

[B12-behavsci-16-01527] Diamond A., Ling D. S. (2016). Conclusions about interventions, programs, and approaches for improving executive functions that appear justified and those that, despite much hype, do not. Developmental Cognitive Neuroscience.

[B13-behavsci-16-01527] Dingemans E., Möhring K. (2019). A life course perspective on working after retirement: What role does the work history play?. Advances in Life Course Research.

[B14-behavsci-16-01527] Duncan J. (2010). The multiple-demand (MD) system of the primate brain: Mental programs for intelligent behaviour. Trends in Cognitive Sciences.

[B15-behavsci-16-01527] Eichenbaum H., Yonelinas A. P., Ranganath C. (2007). The medial temporal lobe and recognition memory. Annual Review of Neuroscience.

[B16-behavsci-16-01527] Eppler-Hattab R., Meshoulam I., Doron I. (2020). Conceptualizing age-friendliness in workplaces: Proposing a new multidimensional model. The Gerontologist.

[B17-behavsci-16-01527] Fattori A., Comotti A., Barnini T., Di Tecco C., Laurino M., Bufano P., Ciocan C., Serra D., Ferrari L., Bonzini M. (2024). Exploring workability in an older working population: Associations with cognitive functioning, sleep quality, and technostress. Frontiers in Public Health.

[B18-behavsci-16-01527] Feng J., Li Q., Smith J. P. (2020). Retirement effect on health status and health behaviors in urban China. World Development.

[B19-behavsci-16-01527] Goodman-Bacon A. (2021). Difference-in-differences with variation in treatment timing. Journal of Econometrics.

[B20-behavsci-16-01527] Guo S., Zheng X. Y. (2023). New evidence of trends in cognitive function among middle-aged and older adults in China, 2011–2018: An age-period-cohort analysis. BMC Geriatrics.

[B21-behavsci-16-01527] Hsu H.-C. (2007). Does social participation by the elderly reduce mortality and cognitive impairment?. Aging & Mental Health.

[B22-behavsci-16-01527] Huang G., Pan Y., Luo Y. (2025). Population-based estimates of healthy working life expectancy in China. BMC Geriatrics.

[B23-behavsci-16-01527] Jia J., Wei C., Chen S., Li F., Tang Y., Qin W., Zhao L., Jin H., Xu H., Wang F., Zhou A., Zuo X., Wu L., Han Y., Han Y., Huang L., Wang Q., Li D., Chu C., Gauthier S. (2018). The cost of Alzheimer’s disease in China and re-estimation of costs worldwide. Alzheimer’s & Dementia.

[B24-behavsci-16-01527] Jia L., Du Y., Chu L., Zhang Z., Li F., Lyu D., Li Y., Li Y., Zhu M., Jiao H., Song Y., Shi Y., Zhang H., Gong M., Wei C., Tang Y., Fang B., Guo D., Wang F., COAST Group (2020). Prevalence, risk factors, and management of dementia and mild cognitive impairment in adults aged 60 years or older in China: A cross-sectional study. The Lancet Public Health.

[B25-behavsci-16-01527] Jiang H. (2026). Socioeconomic reforms are needed to address disparities for the aging rural-to-urban migrant workforce in China. Nature Aging.

[B26-behavsci-16-01527] Jokela M., Ferrie J. E., Gimeno D., Chandola T., Shipley M. J., Head J., Vahtera J., Westerlund H., Marmot M. G., Kivimäki M. (2010). From midlife to early old age: Health trajectories associated with retirement. Epidemiology.

[B27-behavsci-16-01527] Kim J., Park G. R. (2023). Prolonged social isolation and cognitive function in older adults: Lack of informal social contact versus formal social activity as the source of social isolation. Aging & Mental Health.

[B28-behavsci-16-01527] Kobayashi L. C., O’Shea B. Q., Wixom C., Jones R. N., Langa K. M., Weir D., Lee J., Wong R., Gross A. L. (2024). Lifetime occupational skill and later-life cognitive function among older adults in the United States, Mexico, India, and South Africa. Alzheimer’s & Dementia.

[B29-behavsci-16-01527] Kuiper J. S., Zuidersma M., Zuidema S. U., Burgerhof J. G., Stolk R. P., Oude Voshaar R. C., Smidt N. (2016). Social relationships and cognitive decline: A systematic review and meta-analysis of longitudinal cohort studies. International Journal of Epidemiology.

[B30-behavsci-16-01527] Landeiro F., Mughal S., Walsh K., Nye E., Morton J., Williams H., Ghinai I., Castro Y., Leal J., Roberts N., Wace H., Handels R., Lecomte P., Gustavsson A., Roncancio-Diaz E., Belger M., Jhuti G. S., Bouvy J. C., Potashman M. H., ROADMAP Consortium (2020). Health-related quality of life in people with predementia Alzheimer’s disease, mild cognitive impairment or dementia measured with preference-based instruments: A systematic literature review. Alzheimer’s Research & Therapy.

[B31-behavsci-16-01527] Lang I. A., Rice N. E., Wallace R. B., Guralnik J. M., Melzer D. (2007). Smoking cessation and transition into retirement: Analyses from the English Longitudinal Study of Ageing. Age and Ageing.

[B32-behavsci-16-01527] Lenehan M. E., Summers M. J., Saunders N. L. J., Summers J. J., Vickers J. C. (2023). Relationship between education, occupational complexity, and cognitive performance in ageing. Ageing Research Reviews.

[B33-behavsci-16-01527] Li C., Wang L., Ding L., Zhou Y. (2024). Determinants and inequities in healthy working life expectancy in China. Nature Medicine.

[B34-behavsci-16-01527] Li J., Cacchione P. Z., Hodgson N., Riegel B., Keenan B. T., Scharf M. T., Richards K. C., Gooneratne N. S. (2017). Afternoon napping and cognition in Chinese older adults: Findings from the China Health and Retirement Longitudinal Study baseline assessment. Journal of the American Geriatrics Society.

[B35-behavsci-16-01527] Li M., Wang N., Dupre M. E. (2022). Association between the self-reported duration and quality of sleep and cognitive function among middle-aged and older adults in China. Journal of Affective Disorders.

[B36-behavsci-16-01527] Lin L., Cao B., Chen W., Li J., Zhang Y., Guo V. Y. (2022). Association of adverse childhood experiences and social isolation with later-life cognitive function among adults in China. JAMA Network Open.

[B37-behavsci-16-01527] Livingston G., Huntley J., Liu K. Y., Costafreda S. G., Selbæk G., Alladi S., Ames D., Banerjee S., Burns A., Brayne C., Fox N. C., Ferri C. P., Gitlin L. N., Howard R., Kales H. C., Kivimäki M., Larson E. B., Nakasujja N., Rockwood K., Mukadam N. (2024). Dementia prevention, intervention, and care: 2024 report of the Lancet standing Commission. The Lancet.

[B38-behavsci-16-01527] Lu D., Feng X., Ji S. (2025). Post-retirement employment behavior and older people’s expenditure: New evidence from urban China. Ageing & Society.

[B39-behavsci-16-01527] Luo H., Hu H., Zheng Z., Sun C., Yu K. (2024). The impact of living environmental factors on cognitive function and mild cognitive impairment: Evidence from the Chinese elderly population. BMC Public Health.

[B40-behavsci-16-01527] Luo H., Li J., Zhang Q., Cao P., Ren X., Fang A., Liao H., Liu L. (2018). Obesity and the onset of depressive symptoms among middle-aged and older adults in China: Evidence from the CHARLS. BMC Public Health.

[B41-behavsci-16-01527] Lv B., Liang L., Chen A., Yang H., Zhang X., Guo F., Qian H. (2023). Mortality of Alzheimer’s disease and other dementias in China: Past and future decades. International Journal of Public Health.

[B42-behavsci-16-01527] Mein G., Martikainen P., Hemingway H., Stansfeld S., Marmot M. (2003). Is retirement good or bad for mental and physical health functioning? Whitehall II longitudinal study of civil servants. Journal of Epidemiology and Community Health.

[B43-behavsci-16-01527] Ministry of Civil Affairs of China (2025). Statistical bulletin on the development of national undertakings for the aged in 2024.

[B44-behavsci-16-01527] Ministry of Human Resources and Social Security of China (2023). Statistical bulletin on the development of human resources and social security undertakings in 2022.

[B45-behavsci-16-01527] Ministry of Human Resources and Social Security of China (2025). Notice on issuing the interim measures for the implementation of the flexible retirement system: MOHRSS Fa [2024] No. 94.

[B46-behavsci-16-01527] Mizuochi M., Raymo J. M. (2022). Retirement type and cognitive functioning in Japan. The Journals of Gerontology: Series B.

[B47-behavsci-16-01527] National Bureau of Statistics of China (2013). Statistical communiqué of the People’s Republic of China on the 2012 national economic and social development.

[B48-behavsci-16-01527] National Bureau of Statistics of China (2019). Steady growth in population size and significant improvement in population quality: Report No. 20 on achievements in economic and social development since the founding of New China.

[B49-behavsci-16-01527] National Bureau of Statistics of China (2022). China statistical yearbook 2022.

[B50-behavsci-16-01527] National Bureau of Statistics of China (2025). Social undertakings developed steadily and people’s well-being continued to improve: Statistical report on social livelihood since the 14th Five-Year Plan.

[B51-behavsci-16-01527] Oltmanns J., Godde B., Winneke A. H., Richter G., Niemann C., Voelcker-Rehage C., Schömann K., Staudinger U. M. (2017). Don’t lose your brain at work: The role of recurrent novelty at work in cognitive and brain aging. Frontiers in Psychology.

[B52-behavsci-16-01527] Park D. C., Lodi-Smith J., Drew L., Haber S., Hebrank A., Bischof G. N., Aamodt W. (2014). The impact of sustained engagement on cognitive function in older adults: The Synapse Project. Psychological Science.

[B53-behavsci-16-01527] Park Y., Park H., Lee J., Yun B., Yoon J. H. (2025). Association between working after retirement age and lower depressive symptoms among Korean older adults. Journal of Affective Disorders.

[B54-behavsci-16-01527] Rose U., Kersten N., Pattloch D., Conway P. M., Burr H. (2023). Associations between depressive symptoms and 5-year subsequent work nonparticipation due to long-term sickness absence, unemployment and early retirement in a cohort of 2,413 employees in Germany. BMC Public Health.

[B55-behavsci-16-01527] Scarmeas N., Stern Y. (2003). Cognitive reserve and lifestyle. Journal of Clinical and Experimental Neuropsychology.

[B56-behavsci-16-01527] Sewdas R., de Wind A., Stenholm S., Coenen P., Louwerse I., Boot C., van der Beek A. (2020). Association between retirement and mortality: Working longer, living longer? A systematic review and meta-analysis. Journal of Epidemiology and Community Health.

[B57-behavsci-16-01527] Sharma N., Chakrabarti S., Grover S. (2016). Gender differences in caregiving among family caregivers of people with mental illnesses. World Journal of Psychiatry.

[B58-behavsci-16-01527] Shiba K., Kondo N., Kondo K., Kawachi I. (2017). Retirement and mental health: Does social participation mitigate the association? A fixed-effects longitudinal analysis. BMC Public Health.

[B59-behavsci-16-01527] Shiri R., Poutanen J., Haukka E., Härmä M., Ervasti J. (2025). Risk factors for voluntary early old-age retirement in middle-aged workers: A meta-analysis. Scandinavian Journal of Work, Environment & Health.

[B60-behavsci-16-01527] Standing Committee of the National People’s Congress (2024). Decision on implementing the gradual delay of the statutory retirement age.

[B61-behavsci-16-01527] Stern Y. (2009). Cognitive reserve. Neuropsychologia.

[B62-behavsci-16-01527] Stern Y., Barnes C. A., Grady C., Jones R. N., Raz N. (2019). Brain reserve, cognitive reserve, compensation, and maintenance: Operationalization, validity, and mechanisms of cognitive resilience. Neurobiology of Aging.

[B63-behavsci-16-01527] Sun L., Deng G., Lu X., Xie X., Kang L., Sun T., Dai X. (2024). The association between continuing work after retirement and the incidence of frailty: Evidence from the China Health and Retirement Longitudinal Study. The Journal of Nutrition, Health and Aging.

[B64-behavsci-16-01527] Sunderaraman P., Stern Y., Alosco M. L., Stern R. A. (2019). Overview of the reserve concept in the context of cognitive aging. The Oxford handbook of adult cognitive disorders.

[B65-behavsci-16-01527] Takase M., Sugiura K., Nakamoto I., Watanabe S., Murayama H. (2024). The association between employment and cognitive function in older adults: A systematic review. Geriatrics & Gerontology International.

[B66-behavsci-16-01527] Tan N., Chang L., Guo R., Wu B. (2022). The effect of health on the elderly’s labor supply in rural China: Simultaneous equation models with binary, ordered, and censored variables. Frontiers in Public Health.

[B67-behavsci-16-01527] Tur-Sinai A., Shahrabani S., Lowenstein A., Katz R., Halperin D., Fogel-Grinvald H. (2024). What drives older adults to continue working after official retirement age?. Ageing & Society.

[B68-behavsci-16-01527] United Nations (2002). Madrid international plan of action on ageing, 2002.

[B69-behavsci-16-01527] Wan Y. Y., Zeng Y. B., Fang Y. (2021). A study on the effects of labor participation on the health of the retired elderly in China. Chinese Journal of Health Policy.

[B70-behavsci-16-01527] Westerlund H., Kivimäki M., Singh-Manoux A., Melchior M., Ferrie J. E., Pentti J., Jokela M., Leineweber C., Goldberg M., Zins M., Vahtera J. (2009). Self-rated health before and after retirement in France (GAZEL): A cohort study. The Lancet.

[B71-behavsci-16-01527] Westerlund H., Vahtera J., Ferrie J. E., Singh-Manoux A., Pentti J., Melchior M., Leineweber C., Jokela M., Siegrist J., Goldberg M., Zins M., Kivimäki M. (2010). Effect of retirement on major chronic conditions and fatigue: French GAZEL occupational cohort study. BMJ.

[B72-behavsci-16-01527] World Health Organization (2021). Decade of healthy ageing: 2021–2030.

[B73-behavsci-16-01527] Xie L., Yao Y. D., Tang L. L., Zhang S., Yang H. L., Zhang S. Q., Wu Y. Y., Li Z. Y. (2021). Effect of working after retirement on the mental health of older people: Evidence from China. Frontiers in Psychiatry.

[B74-behavsci-16-01527] Zaccagni S., Sigsgaard A. M., Vrangbaek K., Noermark L. P. (2024). Who continues to work after retirement age?. BMC Public Health.

[B75-behavsci-16-01527] Zacher H., Sagha Zadeh R., Heckhausen J., Oettingen G. (2021). Motivation and healthy aging at work. The Journals of Gerontology: Series B.

[B76-behavsci-16-01527] Zhan Y., Wang M., Liu S., Shultz K. S. (2009). Bridge employment and retirees’ health: A longitudinal investigation. Journal of Occupational Health Psychology.

[B77-behavsci-16-01527] Zhang Z., Feng L. (2024). Social participation and its gender differences among ethnic minority elders after poverty alleviation relocation (Linxia, China). Humanities and Social Sciences Communications.

